# Adhesion abilities and biosorption of Cd and Mg by microorganisms - first step for eco-friendly beneficiation of phosphate ore

**DOI:** 10.1038/s41598-019-49406-4

**Published:** 2019-09-10

**Authors:** Hakim Rabia, Malek Ould Hamou, Katarzyna Kasperkiewicz, Jolanta Brożek, Maria Augustyniak

**Affiliations:** 1 Ecole Nationale Polytechnique d’Alger, 10 Avenue Hassen Badi BP 182 El Harrach, 16200 Alger, Algeria; 2 Department of Microbiology, Faculty of Biology and Environmental Protection, University of Silesia in Katowice, Jagiellońska 28, 40-034 Katowice, Poland; 3 Department of Zoology, Faculty of Biology and Environmental Protection, University of Silesia in Katowice, Bankowa 9, 40-007 Katowice, Poland; 4 Department of Animal Physiology and Ecotoxicology, Faculty of Biology and Environmental Protection, University of Silesia in Katowice, Bankowa 9, 40-007 Katowice, Poland

**Keywords:** Soil microbiology, Element cycles

## Abstract

Chemical reagents used in traditional mineral processing can be toxic and hazardous for the environment. Therefore, the use of biotechnological methods is becoming increasingly important. Great hopes are being placed in the use of microorganisms for bio-beneficiation of raw materials. However, assessment of adhesion abilities of bacteria onto minerals surface as well as biosorption of metals are essential steps before designing final process of each ore beneficiation. The main aim of this work was an investigation of biosorption of Cd and Mg, as well as adhesion abilities of five microorganism species with minerals included in the natural mixture of phosphate ore form Djebel Onk, Algeria. The ore, due to its unique composition, created conditions for adhesion of all five tested microbial strains onto apatite surface during incubation at pH 3. Moreover, *Rhodococcus erythropolis* CD 130, *Pseudomonas fluorescens* and *Escherichia coli* adhered distinctly onto apatite surface during incubation at pH 7. Incubation lasting 20 min at pH 4-6 created the most favorable conditions for biosorption of metals by *B. subtilis* and adhesion of cells. In case of *C. albicans*, biosorption of metals as well as adhesion of cells onto the mineral surface were more effective after longer time and in a wider pH range.

## Introduction

In the last decade, cadmium (Cd) contamination of soil became an extremely burning problem for European Union countries. Apart from atmospheric deposition, sewage sludge, compost and manure, the most important source of Cd in soil are phosphate fertilizers used by farmers. Cd, as well as other trace elements, have the ability to accumulate in soil, and can be transferred through food chains, posing a threat to animal and human health^1,2^. Three scenarios of consequences of changing EU policy concerning Cd content in soil, phosphate fertilizers and raw material were presented in the report of the Institute for Environmental Studies, Vrije Universiteit Amsterdam, Netherlands^2^. First of them is rather short-term, and assumes no changes in usage of Cd-rich phosphate ores, with all the financial consequences related to the introduction of a charge for exceeding permissible levels in fertilizers, and thus in the raw material. The second scenario is highly probable, especially in the long-term perspective. It assumes a gradual increase in use of natural low-cadmium raw materials, with all the consequences for countries which have natural deposits of phosphate ores^3^. Third scenario assumes decadmiation of the raw material, allowing phosphate rocks with high initial Cd concentration to be used. However, in the last scenario a new technology has to be developed. Safety of such technology for human health and the environment is crucial^2^. Therefore, particular attention should be paid to biological methods, in which the production of toxic waste during decadmiation would be significantly reduced, while recovery of Cd and other trace elements would be possible. The third scenario can also be promising in the long-term perspective, because global deposits of phosphate rock are limited, like other nonrenewable resources. Sooner or later, industry will have to resort to resources that contain more pollution, including high level of Cd and other trace elements.

Phosphate ore, which can be used as a source material for phosphoric acid, fertilizers, as well as animal feed production, has to fulfill specific requirements. The content of P_2_O_5_ must be higher than 30% of phosphate ore weight. CaO/P_2_O_5_ ratio should be less than 1.6%, and MgO content shouldn’t be higher than 1% of phosphate ore weight^4–7^. Raw materials that do not meet these criteria should undergo beneficiation, during which most of impurities have to be removed^4,7,8^. Depending on the origin of the ore, its’ geological condition, and changes that have occurred after deposition, several minerals can be found in phosphate ore in various proportions^5,8^. Other minerals, like the dolomite, quartz, calcite, clays, or organic deposits can be found in various proportions, together with fluoroapatite, hydroxyapatite, carbonated hydroxyapatite or francolite. These additional minerals are an onerous source of pollution of ore, along with metals like Mg or Cd, that hinder the use of ore in various technological processes and in agriculture. Therefore, one should focus on improving methods to increase efficiency of dolomite removal from phosphate ore.

Chemical reagents used in traditional mineral processing, including froth flotation that require application of frothers, collectors, depressants and other chemicals^4^, can be toxic and hazardous for the environment. Due to the environmental risks posed by conventional methods of ore beneficiation, the use of biotechnological methods is becoming increasingly important. Great hopes are being placed in the use of microorganisms and their metabolic products for bioleaching of metals, but also for biomining and bioremediation^9–12^.

The phenomenon of biosorption of metals by biological materials can be used for mining and metallurgical waste water purification. Potential organic sorbents, such as bacteria^13,14^, fungi^15,16^, and algae^17^, display extracellular accumulation, cell surface sorption or/and intracellular accumulation. The process can be metabolic-dependent or metabolic-independent. Physical adsorption utilizes van der Waals’ forces. Polysaccharides, proteins, lipids and other components that are present on the surface of microorganism cells provide a rich variety of functional groups ready to exchange or bind metal ions. This metabolic-independent process is usually very fast and can be reversible^9^. Accumulation of metals in the cell usually requires more time, and involves transport of ions across the wall. Therefore, a metabolic-dependent process can occur only in living cells^9^.

Another, recently developing field of science is implementation of microorganisms and their metabolites for flotation^11,12^. Microorganisms and/or substances produced by them, such as surfactants^18^, EPS - extracellular polymeric substances^19^, and various proteins^20^ can modify surfaces of various substrates, also minerals. The key task is to match reagents (in classical flotation)^4^, or microorganisms (in bioflotation)^11,12^, to bring about the selective hydrophobization of minerals in a given ore. In their review articles, Behera and Mulaba-Bafubiandi^11^ as well as Dwyer *et al*.^12^ presented several studies, in which applications of microorganism in mineral flotation process have been applied. However, Behera and Mulaba-Bafubiandi point to the need for further detailed research in order to determine the nature and characteristics of bio-reagents responsible for selectively changing the surface of a mineral in a given mixture of ore. Bio-hydrometallurgy is still in a laboratory phase^11^. After studies of adhesion of microorganisms onto pure minerals in a model study, as it was done by Zheng *et al*.^21^ in case of dolomite and apatite, it is the time to test the abilities and properties of microorganisms in a natural mixture of minerals - phosphate ore in case of this study. A large scale production of microbial reagents will become the challenge for the future^11^.

The main aim of this work was an investigation of the interaction of five microorganism species with minerals included in the natural mixture of phosphate ore form Djebel Onk, Kef Essnoun region, Algeria. In preliminary tests, Cd and Mg binding abilities, as well as adhesion onto dolomite or apatite, were tested for five microbial strains. In the second and third tests, bio-sorption and adhesion studies were performed for *Bacillus subtilis* and *Candida albicans*, also in an *in situ* model. Various pH and incubation times were taken into account. The results were discussed in perspective of potential use of tested microorganisms in further bioflotation.

## Results

### Preliminary test results

Cd concentration in control samples (microorganisms incubated without ore) was low, and never exceeded 0.64 µg·g^−1^ (Fig. S1-A). After 20 min of incubation with phosphate ore, Cd accumulation in biomass increased to various degrees, demonstrating differentiation among strains. The highest value was found in *B. subtilis* at pH 7, where it was as high as 13.58 µg Cd·g^−1^ of biomass (Fig. S1-B). Mg accumulation in control samples was pH-dependent (Fig. S1-C). Twenty minutes of incubation with ore resulted in a several-fold increase of Mg content in biomass. The highest values were observed for *C. albicans*, *B. subtilis*, and *P. fluorescens* as well (Fig. S1-D). Ca accumulation in the control samples resembled Mg accumulation (Fig. S1-E). Because *C. albicans* and *B. subtilis* showed relatively high accumulation of all examined elements at pH 7 (which may be beneficial for further work on application of microorganisms in bioflotation) all following research was undertaken on these two strains.

### Metal content in biomass - different time after incubation with ore

**Figure 1:**
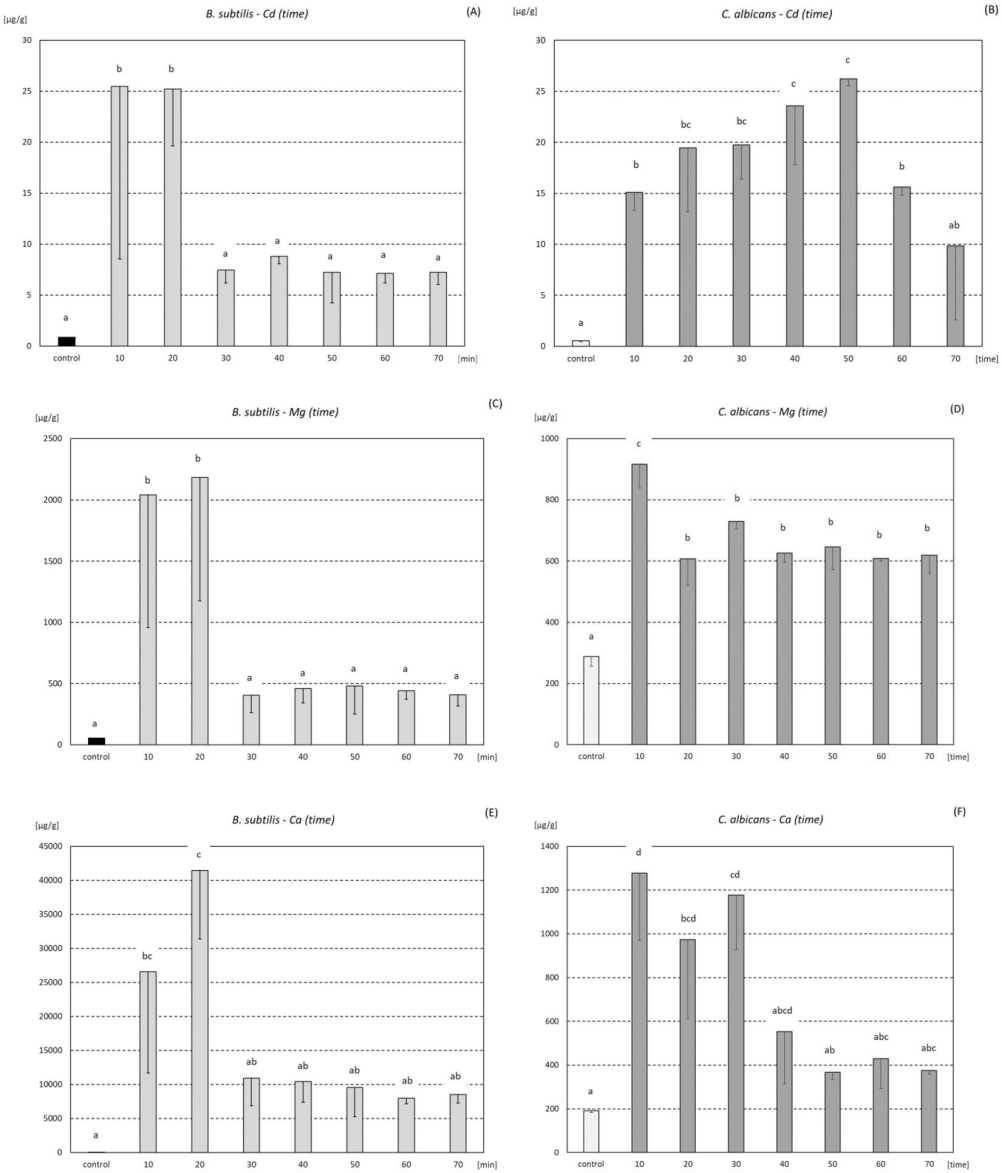
Cd, Mg, and Ca accumulation (mean ± SD; µg·g^−1^) in biomass (*B. subtilis* - **A**,**C**,**E**, and *C. albicans* - **B**,**D**,**F**) after incubation with phosphate ore up to 70 min. The same letter indicate no significant differences among time-groups (ANOVA, LSD; p < 0.05).

Concentration of all elements, measured in biomass of *B. subtilis* after incubation with phosphate ores, revealed a similar pattern. The highest accumulation was found up to 20 min after incubation. Cd concentration in *B. subtilis* cells after 10 and 20 min of incubation was more than 29 times higher than in control group (Fig. 1A, enlarged version in supplementary information in Fig. S1_bis). Starting from 30 min of incubation up to 70 min, the level of Cd accumulation was lower and very stable, oscillating between 7.14 and 8.82 µg·g^−1^. Mg content in *B. subtilis* cells after 10 and 20 min was respectively 38.5 and 41.2 times higher than in control cells (Fig. 1C, Fig. S1_bis). Twenty minutes after incubation, Ca accumulation in *B. subtilis* was the highest. At that time-point the mean value of Ca was 1884 times higher than in control group (Fig. 1E, Fig. S1_bis).

Accumulation of Cd in *C. albicans* increased gradually up to 50 minutes after the end of incubation, reaching a mean value of 26.22 µg·g^−1^ at this time-point. From this point onwards Cd accumulation started to decrease (Fig. 1B, Fig. S1_bis). Mg accumulation in *C. albicans* was about 3 times higher at 10 min after incubation, and about 2 times higher at other time-points, compared to the control group (Fig. 1D, Fig. S1_bis). Incubation with phosphate ore caused significant increase of Ca concentration in *C. albicans* biomass up to 30 min, compared to the control group (Fig. 1F, Fig. S1_bis).

### Metal content in biomass - different pH of incubation

**Figure 2:**
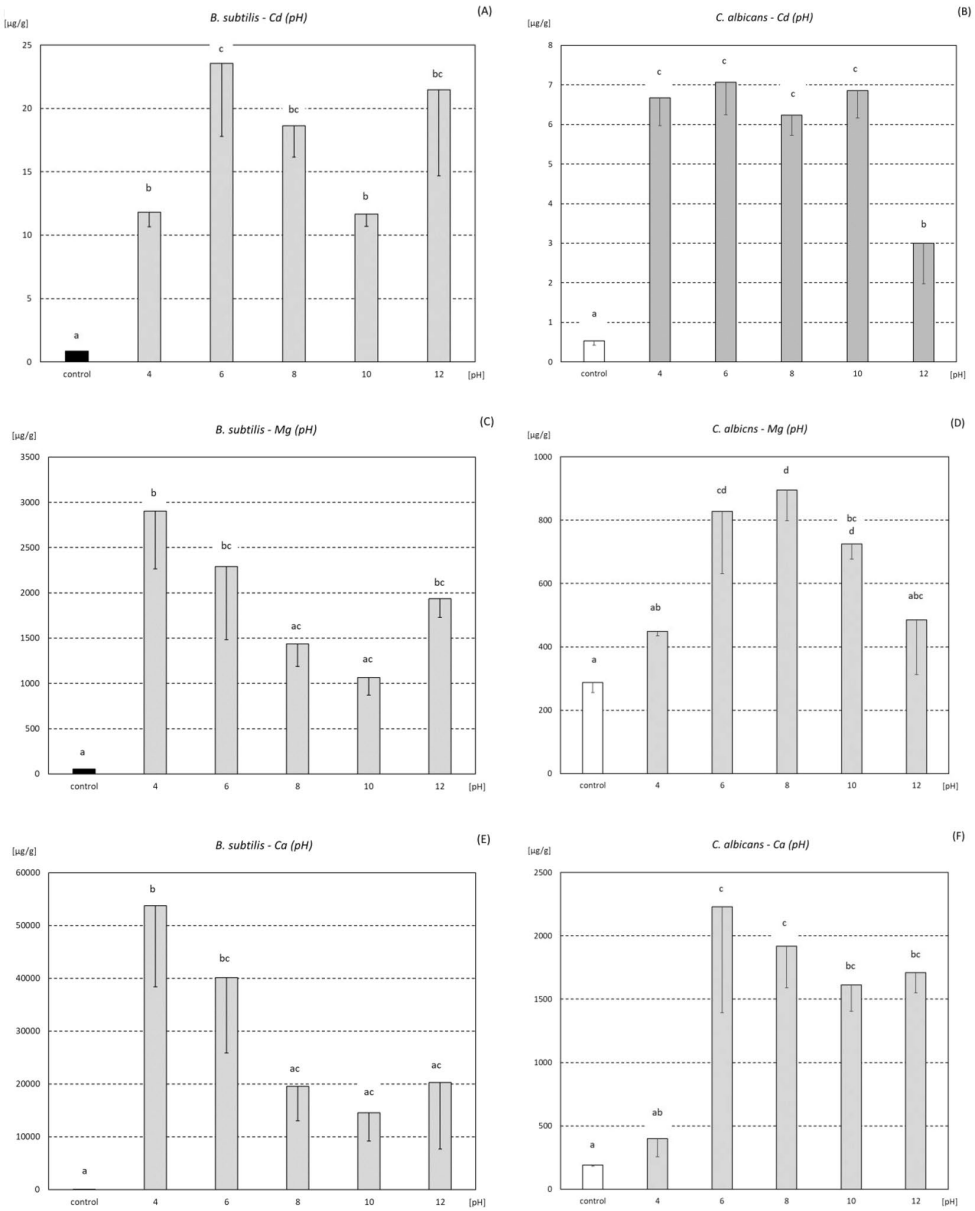
Cd, Mg, and Ca accumulation (mean ± SD; µg·g^−1^) in biomass (*B. subtilis* - **A**,**C**,**E**, and *C. albicans* - **B**,**D**,**F**) after incubation with phosphate ore at different pH. The same letter indicate no significant differences among pH-groups (ANOVA, LSD; p < 0.05).

Concentration of Cd, Mg, and Ca was influenced by pH of solution in which incubation was performed, in case of both tested microorganisms (Fig. 2, Fig. S2_bis). The obtained values were, to a large extent, consistent with the tendency which was found in special control samples (ore incubated without microorganisms). The amount of leached out metals was pH dependent. In higher pH a lower metals content in solution was observed after incubation (Table 1S). The highest Cd accumulation in *B. subtilis* biomass was found at pH 6. Surprisingly, mean value of Cd accumulation at pH 12 was also high, and close to those found at pH 6 and 8 (Fig. 2A, Fig. S2_bis). Cd accumulation in *C. albicans* cells in all tested pH groups was significantly higher than in control cells, and, at pH range between 4 and 10, the mean values created a homogenous group (Fig. 2B, Fig. S2_bis). The highest Mg concentration in *B. subtilis* was found at pH 4 (the mean value was almost 55 times higher than in control group) (Fig. 2C, Fig. S2_bis). *C. albicans* accumulated Mg to a lesser extent than *B. subtilis*. The highest value, which was found at pH 8, was only 3 times higher than in control group (Fig. 2D, Fig. S2_bis). Ca accumulation in *B. subtilis* cells revealed the same pattern as Mg accumulation in this strain (Fig. 2E, Fig. S2_bis). The level of Ca accumulation by *C. albicans* at pH 6 was 11.7 times higher than in the control group (Fig. 2F, Fig. S2_bis). For *B. subtilis* the highest mean Ca content (found after incubation at pH 4) was 2412 times higher than in control group.

### Adhesion/sedimentation of microorganisms onto ore from Djebel Onk

**Figure 3:**
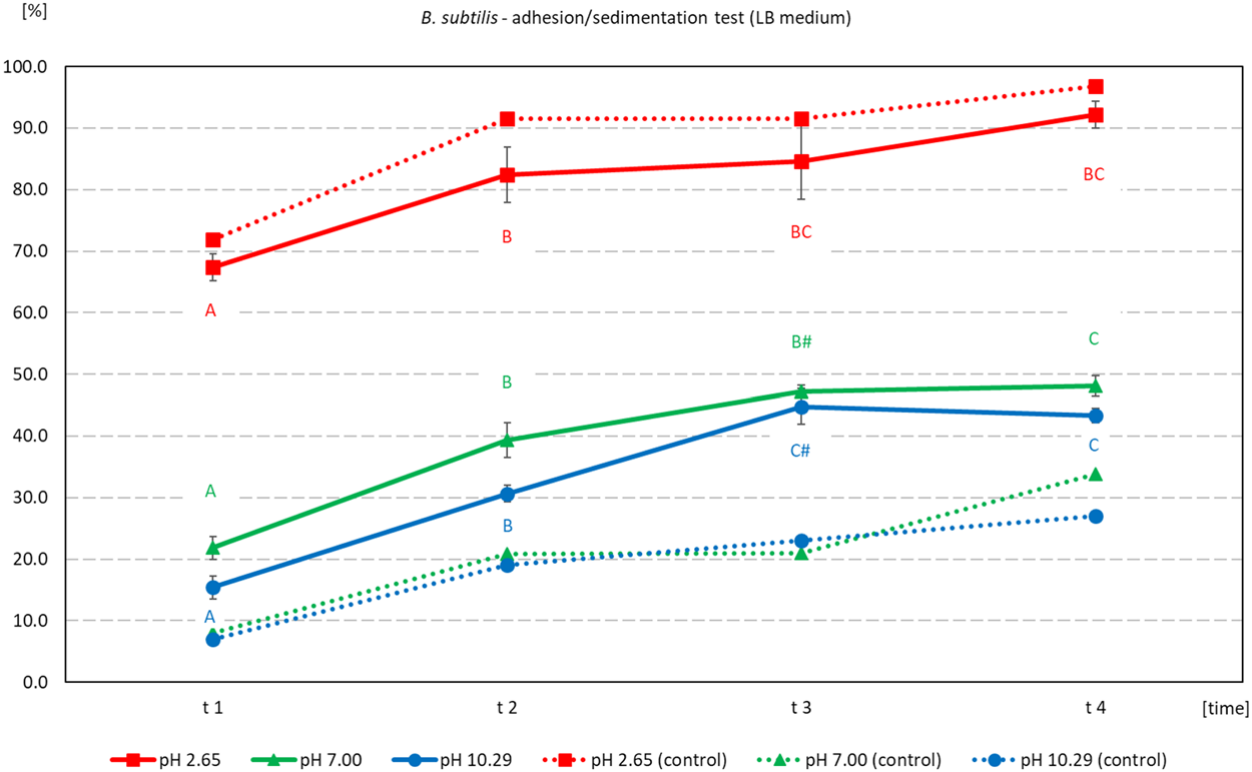
Adhesion/sedimentation [%] of *B. subtilis* cultured in LB media onto Djebel Onk phosphate ore, after incubation at various pH, as a function of time after the end of incubation. Abbreviations: The values were calculated based on optical density (OD) of suspension of bacteria before, and after incubation: % of adhesion/sedimentation = 100% - (initial OD measured before test - final OD measured after incubation with ore at subsequent time-points); solid line - treated groups (bacteria after incubation with ore), dotted lines - control groups (bacteria after incubation without ore); the same letters indicate homogenous time-groups within given pH; the same marks indicate homogenous pH-group within given time. Estimated initial bacteria density = 1.04 × 10^9^ cells/mL.

The process of sedimentation of *B. subtilis* was similar at pH 7 and 10, and was between 7% and 8% at time point t1. Sedimentation in these samples did not exceed 30% until the end of observation (time-point t4). Incubation of bacteria at low pH 2 caused very intensive sedimentation, which reached 72% at time t1. At the end of the experiment, sedimentation at pH 2 reached 97%. Incubation of bacteria with the ore at pH 7 and 10 caused a clearer decrease in OD values, which indicated a higher percentage of adhesion/sedimentation. At time-point t1, the adhesion/sedimentation percentage of *B. subtilis* onto ore was 15.4% at pH 10 and 21.8% at pH 7. After subtracting the percentages of control groups from these values, the observed increase of adhesion was 8.5% and 13.8% at pH 10 and 7, respectively. The values in subsequent time-points were significantly higher compared to these found at time-point t1, which most probably reflects the level of sedimentation in these samples. The level of adhesion/sedimentation in samples incubated with ore at pH 2 was very high, and coincided with values for control samples. This indicates a significant share of independent sedimentation at such low pH (Fig. 3).

**Figure 4:**
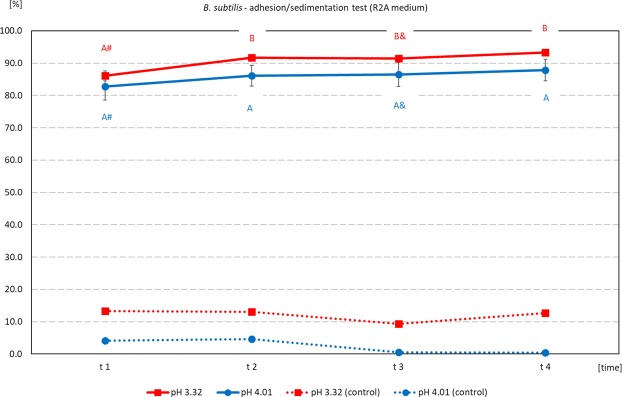
Adhesion/sedimentation [%] of *B. subtilis* cultured in R2A media onto Djebel Onk phosphate ore, after incubation at various pH, as a function of time after the end of incubation. Abbreviations: see Fig. 3. Estimated initial bacteria density = 3.16 × 10^8^ cells/mL.

Because of relatively high sedimentation in the first adhesion/sedimentation test, in the second test a suspension with lower cell density (grown on less nutrient-dense medium) was used. In such conditions, sedimentation in control samples was low and very stable in time (Fig. 4). Adhesion of *B. subtilis* onto phosphate ores from Djebel Onk in these conditions was very high both at pH 3 and 4, oscillating between 82.82% (at t1 time-point in pH 4) and 93.33% (at t4 time-point in pH 3). Adhesion of microorganisms onto ores at pH 4 was stable in time (Fig. 4).

**Figure 5:**
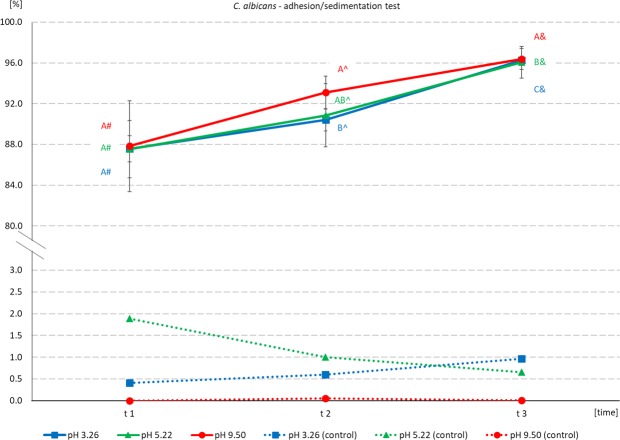
Adhesion/sedimentation [%] of *C. albicans* onto Djebel Onk phosphate ore, after incubation at various pH, as a function of time after the end of incubation. Abbreviations: see Fig. 3. Estimated initial fungus density = 1.78 × 10^9^ cells/mL.

The following values for the number of bacteria that can bind onto 1 g of ore were estimated: pH 10 ~ **0.80 × 10**^**10**^ cells/1 g ore; pH 7 ~ **1.13 × 10**^**10**^ cells/1 g ore, pH 4 ~ **1.29 × 10**^**10**^ cells/1 g ore, pH 3 ~ **1.36 × 10**^**10**^ cells/1 g ore. Incubation of *B. subtilis* with ore, at pH between 3 and 4, results in high adhesion, which is accompanied by relatively low sedimentation. Sedimentation of *C. albicans* in the control samples was low, and similar at all measured pH and all time-points. The values oscillated between 0.007% (at time-point t1 and pH 9) and 1.89% (at time-point t1 and pH 5). Meanwhile adhesion (measured after incubation with ore) was high regardless of pH. Mean values at time-point t1 were very close and equaled: 87.58%, 87.55%, and 87.83% for samples incubated at pH 3, 5 and 9 respectively. Final adhesion (time-point t3) equaled: 96.28%, 96.06%, and 96.39% for samples incubated at pH 3, 5 and 9 respectively (Fig. 5).

**Figure 6:**
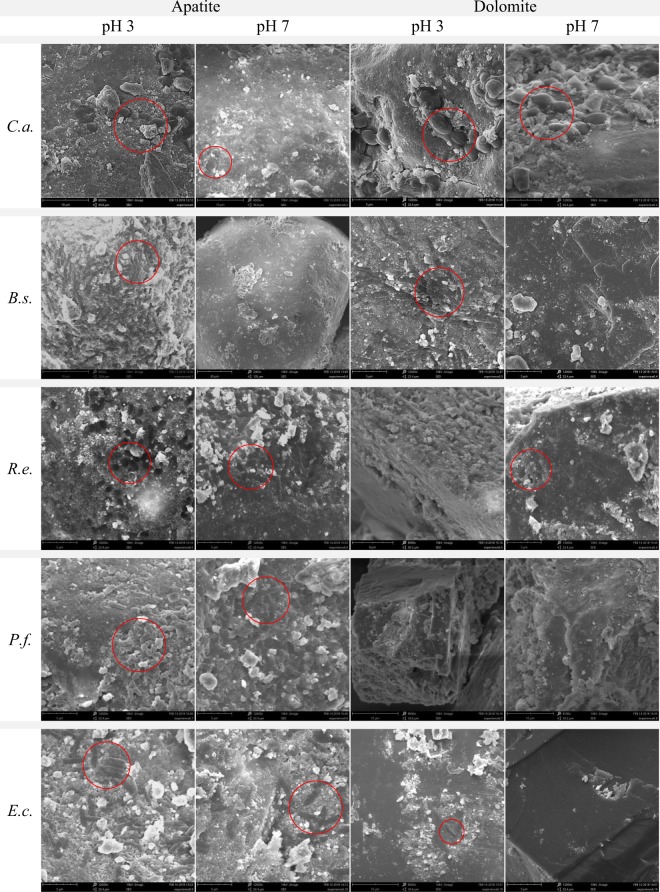
SEM images of apatite or dolomite from Djebel Onk ore, showing various microorganism adsorption onto the minerals surface. Abbreviations: microorganism strains: *Candida albicans* (C.a.), *Bacillus subtilis* (B.s.), *Rhodococcus erythropolis* CD 130 (R.e.), *Pseudomonas fluorescens* (P.f.), and *Escherichia coli* (E.c.) were incubated with ores at different pH (3 or 7), and at 28 °C for 20 min. Red circles - example of microorganisms.

**Figure 7:**
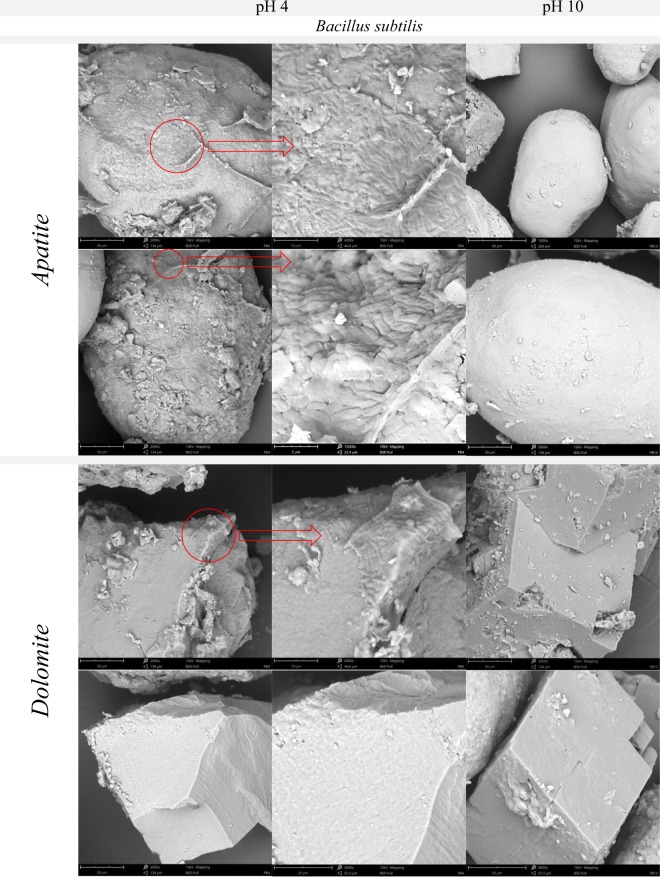
SEM images of apatite and dolomite from Djebel Onk ore, showing *B. subtilis* adsorption onto the minerals surface after incubation at pH 4 or 10 (at 28 °C for 20 min). Red circles - areas which were magnified and showed in the second column.

**Figure 8:**
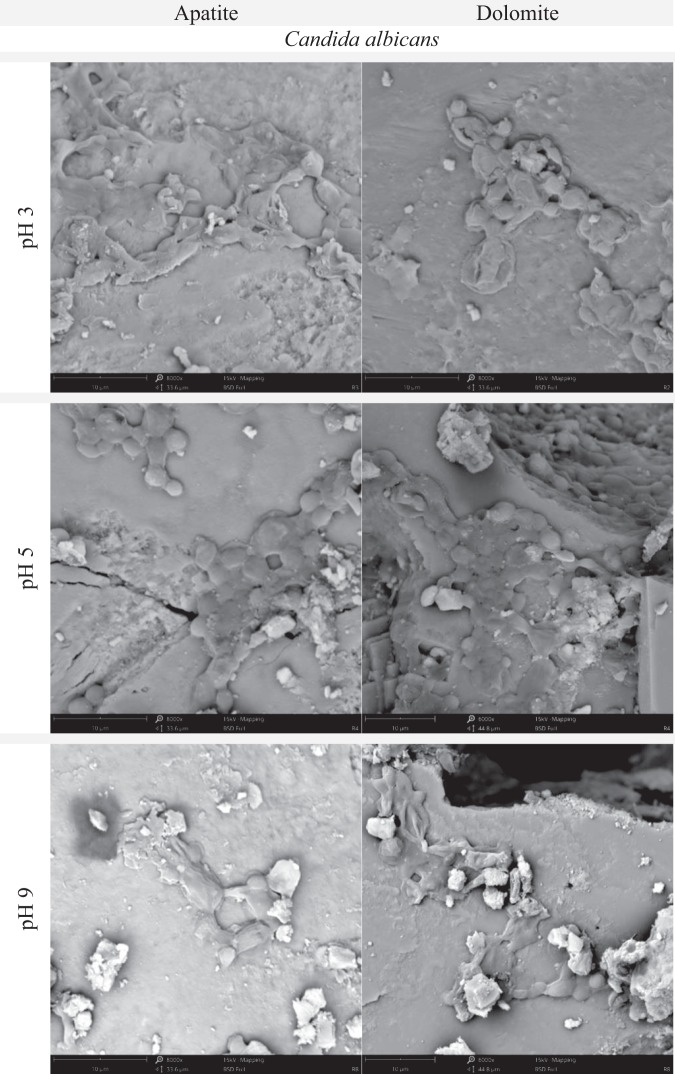
SEM images of apatite and dolomite from Djebel Onk ore, showing *C. albicans* adsorption onto the minerals surface after incubation at pH 3, 5 or 9.5 (at 28 °C for 20 min).

At pH 3 and 4, *B. subtilis* displayed very good adhesion onto apatite particles from Djebel Onk ore (Figs 6–8, Figs S6–S8). High resolution images allow to assume that the adhesion of *B. subtilis* onto surface was strong (Fig. S2). The adhesion of *B. subtilis* onto dolomite at pH 3–4 was also observed, but to a lesser degree than in case of apatite (Figs. 6–8, Figs S6–S8). As it was in case of apatite, adhesion of bacteria onto dolomite seemed to also be very strong (Fig. S2). At pH 7 and 10, adhesion of *B. subtilis* onto particles, both apatite and dolomite, was limited only to loosely spaced, single bacterial cells, but mostly - adhesion was not observed.

*C. albicans* colonized dolomite particles slightly more efficiently than apatite particles. In the first test, many *C. albicans* cells were found on dolomite particles at pH 3 and 7 (Fig. 6, Fig. S6). In the next test, the level of colonization by *C. albicans* was the highest at pH 5, in case of both apatite and dolomite (Fig. 8, Fig. S8). At pH 9 the cells were also found on the surface of minerals, but to a much lesser degree. In turn, *R. erythropolis* CD 130 colonized apatite particles to a higher extent, and was observed on the surface of apatite after incubation at pH 3 and 7. Occasionally, cells of *R. erythropolis* CD 130 were observed on the surface of dolomites after incubation at pH 7. *P. fluorescens* displayed good colonization of apatite particles and weak adhesion to dolomite particles in both tested pH values. *E. coli* was also more likely to colonize apatite particles, and displayed almost no adhesion to dolomite particles (Fig. 6, Fig. S6).

## Discussion

The screening test showed that the content of Cd and Mg in biomass after incubation with phosphate ore from Djebel Onk increased for all tested microbial strains (Fig. S1). However, two out of five strains, namely *B. subtilis* and *C. albicans*, appeared to be particularly promising. In subsequent tests we showed that *B. subtilis* binds metals in a higher amount during up to 20 minutes of incubation (Fig. 1, Fig. S1_bis), which is consistent with the literature data. The rapid change of metal concentration in biomass, that we observed in our experiment (Fig. 1, Fig. S1_bis), suggests the biosorbtion phenomenon, which involves mechanisms of binding metals onto the surface of a cell, and not bioaccumulation, where the transport of ions into the cell takes place. To explain the observed results, Van der Waals forces, together with ion exchange and/or complexation with compounds presented in the microbial cell wall should be considered^22^. Assuming that the rapid metal accumulation observed by us (Fig. 1, Fig. S1_bis) can be an effect of biosorption (metabolic-independent process^9^) inactivated cells can be used for further tests, making biomass safe for use.

Bacteria are characterized by high surface to volume ratio and offer an abundance of functional groups that can participate in biosorption. To date, hydroxyl, carboxyl, amino, ester, sulfhydryl, carbonyl, and phosphoryl groups were identified as functional groups involved in biosorption^23^. X-ray absorption fine structure (XAFS) spectroscopy analysis indicated that below pH 4.4, Cd binds mostly to phosphoryl ligands present in the *B. subtilis* wall, whereas at higher pH, adsorption to carboxyl groups becomes more important. At pH 7.8, activation of an additional binding site, described as a phosphoryl site (with smaller Cd-P distance than the one that is active at lower pH), was observed^24^. Most probably, the phenomena described above may be the cause of differences observed by us in the biosorption of metals (Fig. 2, Fig. S2_bis), that was pH dependent. Essential and non-toxic elements, such as Mg or Ca, can be exchanged by other ions of toxic elements (e.g. Cd). Endo and Aoyagi^14^ reported the maximum adsorption of divalent metal ions by *Lactobacillus casei* JCM1134 in the following order: Cu^2+^ > Ba^2+^ > Sr^2+^ > Cd^2+^ > Co^2+^ > Mg^2+^ > Ni^2+^. The authors found high positive correlation of ions adsorption with the ionic radius and postulated that metal adsorption by *L. casei* JCM1134 was greatly realized by an ion exchange mechanism^14^. However, biogenic elements, such as Mg, Ca, Zn or Cu, are also biosorbed (Figs 1 and 2, Figs S1_bis and S2_bis). The basis of this phenomenon can be metal complexation, as it was found in the case of *Pseudomonas syringae*^9,22^. Biosorbtion observed in our experiments was reversible, thus, most likely, Van der Waals forces and/or the Coulombic Attraction forces were involved. The range of Cd biosorption that we observed for microorganisms was not very high (Figs 1 and 2, Figs S1_bis and S2_bis). Even *R. erythropolis* CD 130, for which siderophore secretion was proved^25^, did not bind large amounts of Cd (Fig. S1). At this point, it should be clearly stated that the Cd content in our samples, and thus in the solution obtained after leaching elements out of the ore (Table S1), was not as high as in the case of other industrial wastewater, which arose as a result of direct contact with Cd-rich materials. Estimated calculations showed that the amount of bacteria used during the experiments (about 0.2–0.3 g of dry weight·L^−1^) allowed for biosorption of a vast majority (or even all) of leached metals. Thus, a 20-minute incubation with bacteria can effectively reduce Cd from wastewater that was generated after phosphate ore treatment. However, further detailed research and more sofisticated calculations should be porformed. Cd sorption by resistant/tolerant bacteria can be significantly higher^26–29^. Gourdon *et al*.^28^ evaluated Cd biosorption abilities in Gram-positive and Gram-negative bacteria isolated from sludge, and showed that biosorption of Cd in both groups of bacteria was pH-dependent. Gram-positive bacteria revealed higher Cd biosorption abilities than Gram-negative bacteria^28^. Bacterial biosorption of essential (but harmful) or nonessential (and toxic) metals was also described or reviewed by others^9,22,23^. Also, a variety of fungal species have a great potential to bind metals (Figs 1 and 2, Figs S1_bis and S2_bis). Chitin, which is present in the cell wall, offer amino, amide and hydroxyl groups that assist sorption of metals^22,23,30,31^.

The amount of metals leached out from the sample of ore (after incubation without any microorganism) was pH dependent (Table 1S), and certainly influenced the biosorption dynamic, especially in case of *B. subtilis* which reached higher values of metal content. One can suspect that biosorption for this species is therefore limited by higher pH, which is directly related to the availability of metals in the solution. In sum, biosorption depends on pH, type of metal ions, concentration and properties of biomass, but also pretreatment of biomass, and presence of various ligands in solution^31^. In our experimental conditions, using phosphate ore from Djebel Onk, we found that biosorbtion was species-, time- and pH-dependent (Figs S1 and 1–2). Content of Mg in *B. subtilis* biomass was about two times higher than in *C. albicans* cells. Moreover, Ca biosorption by *B. subtilis* was about 40 times higher than sorption of Ca by *C. albicans* cells (Fig. 1, Fig. S1_bis). Explanation of the results can be connected with the specific conditions that were present during incubation with phosphate ore from Djebel Onk, as well as distinct demand for ions of both microorganisms. *C. albicans* cells are larger than *B. subtilis*, which means that the total area of sorption (calculated for a given mass) for *C. albicans* is smaller. Also the amount and quality of functional groups that are present in chitin^22,23,30,31^ can be less efficient than those present in *B. subtilis* surface. Ca ions were widely available in the incubation mixture, as they are present in dolomite and apatite. Mg ions are a component of dolomites^5^. Certainly, such composition determines and influences adhesion efficiency of these microorganisms to particular components of phosphate ore, in the specific conditions that we set in our experiment. Only the combination of results of biosorption and adhesion can give a full view of the behavior of microorganisms under the special conditions caused by ore.

We found that adhesion of *B. subtilis* onto phosphate ore form Djebel Onk was effective at pH between 3 and 4 and was stable after the end of incubation (Fig. 4). Meanwhile, *C. albicans* revealed very good adhesion over a wide pH range (Fig. 5). There are not many articles discussing the problem of adhesion of microorganisms onto minerals present in phosphate ore. All of these new articles prove that the use of bacteria as bioreagents in phosphate ore processing is still in early stages of development^21,32–37^. Zheng *et al*.^21^ studied adhesion of two bacteria, *Bacillus subtilis* and *Mycobacterium phlei*, onto dolomite and apatite, and found that both bacteria adhered to dolomite better than to apatite (at acidic and near neutral pH values). The authors explain that a wall of Gram-positive bacteria can be perceived as “a layer of microporous ion exchanger”. Furthermore they report that the amount of Mg^2+^ which can be bound by *B. subtilis* is about 10 times greater than Ca^2+^. Thus, *B. subtilis* should adhere more strongly to minerals containing Mg^2+^ (e.g. dolomite), than to minerals that do not contain this element (e.g. apatite). This valuable research, however, was done on high quality of dolomite (20.62% MgO) and apatite (0.07% MgO), and adhesion tests were most probably done separately for each mineral^21^. However, we cannot ignore the fact that bacteria can also bind Ca ions, which are present in both dolomite and apatite. In our experiment, designed for phosphate ore with natural composition of dolomite and apatite (*in situ* approach), SEM analysis revealed better adhesion of *B. subtilis* onto apatite at pH 3–4, than for dolomite (Figs 6 and 7, Figs S6–S7 and S2). *C. albicans* presented good adhesion to both apatite and dolomite, at all tested pH values (Figs 6 and 8, Figs S6 and S8). Good and selective adhesion of *R. erythropolis* CD 130, *P. fluorescens* and *E. coli* to apatite at both tested pH (namely 3 and 7), together with almost complete lack of adhesion to dolomite (Figs 6, S6), shows a lot of potential. These strains will be tested in the future by us.

Certainly, the incubation time and cell concentration are important for the adhesion of bacteria onto substrate surface. At very high bacteria concentration some interaction between cells and increased sedimentation can occure (Figs 3 and 4). After sufficient time, a biofilm formation process will start on substrate surface^20,38–41^. Divalent cations, such as Mg^2+^ and Ca^2+^, can have an important impact on biofilm formation. Recent research has shown that Mg ions can slow down the formation of *B. subtilis* biofilm at Mg^2+^ concentration of 50 mM or higher, but do not inhibit growth of bacteria themselves. Most probably, Mg^2+^ inhibit expression of genes responsible for extracellular matrix formation^38^. On the other hand, there are studies showing that the presence of Ca ions can restrict *B. subtilis* biofilm expansion. In the absence of Ca^2+^ in the media, peripheral cells of the colony can expand faster, and boost the size of the colony^42^.

Adhesion of the microorganisms onto the mineral surface is one of the most important steps in mineral bioprocessing^32,33,36^. This is because microorganisms modify the surface of minerals, and therefore separation of minerals is possible. Merma *et al*.^33,36^ report that *Rhodococcus opacus* seems to be useful for separating apatite, calcite and quartz particles under specific conditions. Analysis of SEM images confirmed that *R. opacus* tends to adhere onto surfaces of both apatite and quartz, but the level of adhesion onto apatite is greater. According to Smith and Miettinen^37^, *Saccharomyces carnosus* can interact with surfaces of apatite, calcite and quartz. Both alive microorganisms and freeze dried cells improved recovery of appetite during tests conducted at pH 9. Last year, commercial baker’s yeast cells (BYC - *Saccharomyces cerevisiae*) were also tested^32^. The authors demonstrated yeast adhesion to high-purity apatite crystals at a wide pH range. Reduction of pH from 10 to 7 during mineral processing seems to be very attractive for the future, as it can diminish costs of mineral beneficiation and limit the detrimental impact of mineral processing on the environment. However, as the authors claim themselves, more tests with phosphate ore are needed to prove the potential use of BYC as an industrial bioreagent^32^. Egyptian scientists also work on the use of bacteria to improve phosphate ore processing^34,35^. They observed that *Corynebacterium diphtheria intermedius* and *Pseudomonas aeruginosa* can modify dolomite and phosphate surfaces and improve the selectivity of separation. *P. aeruginosa* gave better results than *C. diphtheria intermedius*. In addition, longer incubation time of minerals with bacteria effectively reduced the MgO content to below 1%^34^. Abdel-Khalek *et al*.^35^ isolated *Desulfvibrio desulfuricans* from phosphate ore and used for tests of separation of silica from apatite. Bacteria revealed better affinity to apatite than quartz surface. However, a separation test of natural phosphate ore with *D. desulfuricans*, conducted at pH 3, allowed them to acquire a concentrate containing 30% P_2_O_5_ and 10% SiO_2_ from slurry containing 20.52% P_2_O_5_ and 23.51% SiO_2_^35^.

Undoubtedly, each deposit is characterized by unique composition and properties that create particular conditions for microorganisms. It is unlikely to develop one, universal method for ore bioprocessing. Therefore, each phosphate rock should be treated individually for the most effective bioprocessing. Detailed biosorption and adhesion tests should always be performed before attempting to use microorganisms for beneficiation of a given ore.

## Material and Methods

### Material characteristic and sample preparation

The samples, originated from the Djebel Onk mine, are representative for the entire Kef Essnoun region. Raw material was obtained courtesy of the National Company of Iron and Phosphate FERPHOS. Mechanical preparation of the ore included crushing, grinding and granulometric study. The particle size analysis of the sample revealed that 3% of the particles were less than 80 μm. Subsequent mechanical preparation involved reduction of fine particles and collection of desirable size of grains during grinding (Fig. S3).

Particles between 80–160 μm in size were used for metal accumulation and adhesion tests. Mineralogical composition of Djebel Onk phosphate ore of 80–160 μm fraction was as follows: carbonate fluoroapatite (CFA; Ca_5_(PO_4_,CO_3_)_3_F) 61.6%, dolomite (CaMg(CO_3_)_2_) 29.1%, calcite (CaCO_3_) 1.1%, clinoptilolite (Ca_2-3_[Al_3_(Al,Si)_2_Si_13_O_36_]·12H_2_O) 2.1%, and quartz (SiO_2_) 6.1%. Cd and Mg concentration in the sample was 36.8 and 5369.1 mg·kg^−1^ (detailed mineralogical and elemental composition of each of the 16 size-class fractions, determined by XRD and AAS methods, are currently being prepared for publication). Before all tests, ore was sterilized to eliminate any microorganisms interfering with the experiment. Samples of 1 g of ore were weighed out, autoclaved (121 °C, 2 h), and stored in sterile conditions for further use.

### Microorganism strains and growth conditions

Microorganisms used in the experiments were obtained from the microbial collection of the Faculty of Biology and Environmental Protection, University of Silesia in Katowice, Poland. For tests of metal adsorption and microbial adhesion onto ore, strains of five microorganisms were selected, based on literature studies and prior experience of microbiologists from University of Silesia. Four microbial strains: *Bacillus subtilis*, *Rhodococcus erythropolis* CD 130, *Pseudomonas fluorescens*, and *Escherichia coli*, as well as one fungal strain: *Candida albicans* were chosen. Strain of *B. subtilis* and *R. erythropolis* CD 130 belong to Gram-positive, while *P. fluorescens*, *E. coli* to Gram-negative bacteria. *R. erythropolis* CD 130 strain was isolated by Magdalena Pacwa-Płociniczak *et al*.^25^ from soil heavily contaminated with petroleum hydrocarbons and characterized by high phosphate solubilization activity and siderophore production^25,43^.

Depending on the type of experiment, one of two types of medium was used for bacteria cultivation. As a rich medium Luria-Bertani broth (LB) (10 g·L^−1^ peptone K, 10 g·L^−1^ sodium chloride, and 5 g·L^−1^ yeast extract) was chosen. As a medium with low nutrient concentration (nutrient-poor) we used R2A medium (0.5 g·L^−1^ yeast extract, 0.5 g·L^−1^ proteose peptone, 0.5 g·L^−1^ peptone K, 0.5 g·L^−1^ glucose, 0.5 g·L^−1^ starch, 0.3 g·L^−1^ dipotassium phosphate, 0.024 g·L^−1^ magnesium sulfate, 0.3 g·L^−1^ sodium pyruvate, 15 g·L^−1^ agar). *Candida albicans* were cultivated in broth with glucose (0.5 g·L^−1^ peptone, 2.0 g·L^−1^ yeast extract, 2.0 g·L^−1^ meat extract, 4.0 g·L^−1^ sodium chloride, 20.0 g·L^−1^ glucose, 20.0 g·L^−1^ agar). The components were purchased from BTL sp. z o.o. company.

Cultivation of bacteria in LB medium and *C. albicans* in broth with glucose was performed at 28 °C on a rotary shaker at 120 rpm for 24 h. In case of microorganisms grown in R2A medium, to get a sufficient number of bacteria, they were cultured up to 72 h. Then, the cultures were centrifuged at 5000 rpm for 15 min, and biomass was washed with 0.98% NaCl three times. Finally, the cells were resuspended in 0.98% NaCl and, for assessment of the initial number of microorganisms, spectrophotometric density was measured at 600 nm using a double beam UV spectrophotometer (ThermoSpectronic).

### Experimental models

#### Experiment 1

In the first experiment (screening test), the five strains of microorganisms were checked for their ability to accumulate metals and adsorb onto surface of minerals. For this experiment selected bacteria were cultivated in LB medium for 24 h, then washed, centrifuged and suspended in 0.98% NaCl. To standardize the approximate number of bacteria in a suspension McFarland standards were used. Wherever necessary, the suspensions were diluted with 0.98% NaCl. The sterilized 1 g samples of ore were incubated (at 28 °C with continuous shaking) with microorganism suspension (50 mL), at various pH and constant time (pH 3 and 7; 20 min). Immediately after incubation, samples were left for 5 min to allow for particle sedimentation. Microorganism suspension was gently decanted into 50 mL Faclcon tubes. Then, a small amount (~0.1 g) of ore was taken and prepared for SEM analysis. The rest of ore was washed with 0.98% NaCl (5 mL) and, after decantation, the liquid with detached cells was added to microorganism suspension. The whole suspension was centrifuged (5000 rpm, 15 min) and the obtained biomass was gently washed with deionized water, lyophilized and prepared for determination of metal content by AAS method.

#### Experiment 2

In the second experiment, two species of microorganisms, *B. subtilis* and *C. albicans*, were selected based on the results of the first test. *B. subtilis* and *C. albicans* revealed a relatively high levels of metal accumulation and adhesion onto dolomite particles. The aim of the experiment was to establish optimal conditions (pH and time) for incubation of microorganisms with ore, in order to maximize metal accumulation in biomass and/or adhesion of cells onto minerals. In the first part of the experiment the sterilized samples of 1 g of ore were incubated with *B. subtilis* or *C. albicans* suspended in 50 mL of 0.98% NaCl (28 °C, shaking: 120 rpm, pH 7) in conical flasks. Times of incubation were as follows: 10, 20, 30, 40, 50, 60, and 70 minutes. In the second part of the experiment the conditions remained the same except for pH, which was variable. The incubation was carried out for 20 min at pH: 4, 6, 8, 10, and 12. Immediately after incubation the ore were sedimented (5 min), then the microorganism suspension was decanted and centrifuged (at 5000 rpm for 15 min). Biomass and minerals were prepared for AAS and SEM analysis as described previously. Control samples consisted of microorganisms incubated at pH 7 for 20 min. All experimental groups contained 4 technical replications. Special control, involving samples of ore incubated without microorganisms at various pH, were also performed. This way the amount of leached out metals, at a given experimental conditions, was estimated (Table S1).

#### Experiment 3

Third experiment included one test for *C. albicans* and two tests for *B. subtilis*. Second test for *B. subtilis* was designed after receiving results of the first test. In the second test pH was limited to the promising range (3–4) as well as the amount of microorganisms was lower than in the first test. *B. subtilis* was cultured in LB (first test) and R2A (second test) media. After cultivation microorganisms were recovered from the media, washed three times and suspended in 0.98% NaCl. Total suspensions were divided into three (or two) portions and pH was adjusted. For bacteria cultured in LB medium, pH 2, 7, and 10 was established. For bacteria cultured in R2A a pH of medium was established as followed 3 and 4. For *C. albicans* pH 3, 5, and 9 was established. Next, all suspensions were divided into 50 mL samples, and 1 g of sterilized ore was added to each. Incubation was carried out in standard conditions: 28 °C with continuous shaking (120 rpm) for 20 min. The level of adhesion was evaluated based on optical density (OD) after pH adjustment. Then the ore was added to bacterial suspension and OD was measured again in four time-points after incubation. The following time-points were established for *B. subtilis*: t1 – 10 min, t2 – 20 min, t3 – 30 min, and t4 – 40 min after the end of incubation. For *C. albicans* OD was measured at t1 – 10 min, t2 – 20 min, and t3 – 30 min after the end of incubation. In the end, minerals were separated by sedimentation and prepared for SEM analysis. Control samples consisted of microorganisms incubated in the same conditions, but without ore. All experimental groups consisted of 4 technical replications, while control groups were performed with two replications.

### Cd, Mg and Ca content assessment

After incubation experiment with ore, the suspensions of microorganisms were centrifuged (Ultracentrifuge Beckman Optima LE-80K) at 8000 rpm and 10 °C for 15 min. Then, biomass was frozen to −70 °C and lyophilized (Freeze dryer Alpha 1–4; Christ, Germany) at −35 °C and pressure 0.2 mBar for 24 h. A portions of ~0.02 g dried microorganisms were mineralized with 0.5 mL of ~65% HNO_3_ at 110 °C for 48 h. After mineralization was completed, the samples were diluted with deionized water to a total volume of 5 mL. Cd, Mg and Ca contents were measured by AAS methods with an iCE™ 3500 AAS atomic absorption spectrometer (Thermo Fisher Scientific). Quality of the analytical procedure was confirmed using standard solutions from Merck at initial concentration of 1 g of metal·L^−1^ of water. Metal content was expressed as µg·g^−1^ of biomass. The metal analyses for samples in the screening experiment (Exp. 1) and control samples in subsequent experiments were done in two replications. The test for metal content in biomass after incubation with ores in each experimental group was replicated for 4 samples.

### Particle surface analysis

After incubation with microorganisms, the ore samples were frozen to −70 °C and lyophilized (Freeze dryer Alpha 1–4; Christ, Germany) at −35 °C and pressure 0.2 mBar for 4 h. After drying, the ores were gently powdered, stuck to an aluminum cylinder with a double sided adhesive carbon tape and coated with ≥ 15 nm film of gold in Pelco SC6 coater (Ted Pella, Inc.). The gold layer covering the sample reduced adverse effects (charging, thermal damage) related to the electron beam action in microscopic vacuum, and improved the imaging of samples. All samples were imaged using Scanning Electron Microscopy Phenom XL at 15 kV accelerating voltage. For better resolution, selected samples (See Fig. S2) were also photographed using a Hitachi SU8010 (FESEM; Hitachi High-Technologies Corporation, Tokyo, Japan). Imaging was performed in the scanning microscopy laboratories of the Faculty of Biology and Environmental Protection of the University of Silesia in Katowice.

### Adhesion/sedimentation calculation

Adhesion and sedimentation levels were calculated based on optical density (OD) measured at wavelength 600 nm, in suspension of bacteria before and after incubation. The following formula was applied:

$$ \% \mathrm{Sedimentation}=\mathrm{100} \% -[({\rm{OD}}c\ast \mathrm{100} \% )/\mathrm{OD}i]$$

$$ \% {\rm{Adhesion}}=100 \% -[({\rm{OD}}o\ast \mathrm{100} \% )/\mathrm{OD}i]$$

where: OD*i* - initial optical density, before mixing bacteria suspension with ore; OD*c* - optical density in control samples (without ore) after incubation; OD*o* - optical density in suspension after incubation with ore.

Estimated number of bacteria that can bind onto 1 g of ore was calculated based on OD*o* - OD*i* and assumption: OD_600_ of 1.0 = 8 × 10^8^ cells/mL. Assuming the value obtained at time-point t1 is the predominant effect of adhesion, and taking the difference between the initial and t1 OD values into account, the number of bacteria that was bound on the surface of 1 gram of 80–160 μm particles was estimated. The decrease in optical density (OD) value in the first test was: 0.874, 0.283, 0.200 at pH 2, 7, and 10, respectively. In the second test, the difference between the initial value and the one measured at time-point t1 was 0.340 and 0.323 at pH 3 and 4, respectively. Because of extremely high sedimentation obtained at pH 2 this data were omitted during estimation of number of bacteria that can bind onto 1 g of ore.

Dotted lines in Figs 3–5 represent percentage values based on optical density (OD) of the suspension. They were measured in control samples after incubation of microorganisms without ore, and reflect the level of sedimentation.

### Statistical analysis

In the first test (screening study), metal concentrations in biomass of five microorganisms were measured in two replications for each group. Therefore, in Fig. S1 the mean of two measurements has been presented, without standard deviation. All other analyses (in the second and third experiments) were done in four replications, and thus statistical procedures have been applied. The rare outlying results, identified by means of Q-Dixon test, were omitted prior statistical analysis. The normality of data was tested using the Kolmogorov-Smirnov and Shapiro-Wilk tests. The homogeneity of variances was checked using the Levene test. Parameters fulfilled the criteria for normal distribution and variance homogeneity. Consequently, parametric tests were used to evaluate the significance of differences among the experimental groups. Accumulation of metals and adhesion of microorganisms onto ore were expressed as mean ± SD. Analysis of variance was performed, and Tukey honest significant difference HSD test (ANOVA; p < 0.05) was used to identify differences separately for each strain and for metal content or pH (in the adhesion test). Statistical analysis was conducted using Statistica 13.1 software.

## Supplementary information

Supplementary information
