# Supplementary material for: Adhesion abilities and biosorption of Cd and Mg by microorganisms - first step for eco-friendly beneficiation of phosphate ore
Source: Sci Rep. 2019 Sep 10;9:12929. doi: 10.1038/s41598-019-49406-4 (PMC6737029; doi:10.1038/s41598-019-49406-4)

Adhesion abilities and biosorption of Cd and Mg by microorganisms

- first step for eco-friendly beneficiation of phosphate ore

Hakim Rabia[1], Malek Ould Hamou[1], Katarzyna Kasperkiewicz[2], Jolanta Brożek[3], Maria Augustyniak[4*]

[1]Ecole Nationale Polytechnique d'Alger, 10 Avenue Hassen Badi BP 182 El Harrach 16200 Alger, Algérie; [2-4]Faculty of Biology and Environmental Protection, University of Silesia in Katowice, Jagiellońska 28, 40-034 Katowice, Poland ([2]Department of Microbiology, [3]Department of Zoology; [4]Department of Animal Physiology and Ecotoxicology)

Fig. S1. Preliminary test results. Cd, Mg and Ca concentration (µg·g^{-1}) in five microorganism strains: *Candida albicans*, *Bacillus subtilis*, *Rhodococcus erythropolis* CD 130, *Pseudomonas fluorescens*, and *Escherichia coli*, that were incubated without (control samples - A, C, E) or with ores (treated samples - B, D, F) at 28 °C and different pH, for 20 min. ND - not detected.

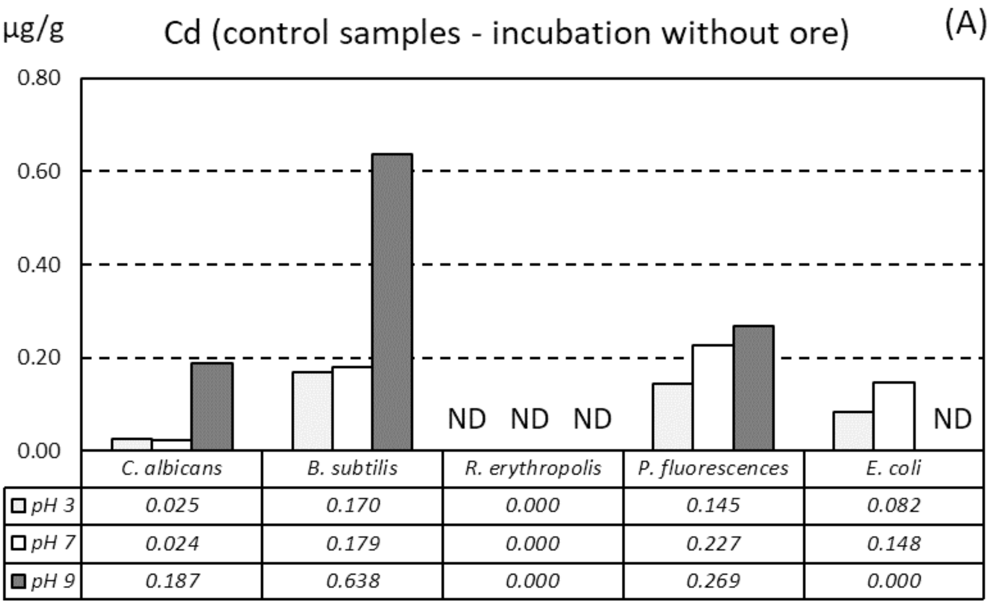

	C. albicans	*B. subtilis*	*R. erythropolis*	*P. fluorescences*	*E. coli*
pH 3	*0.025*	*0.170*	*0.000*	*0.145*	*0.082*
pH 7	*0.024*	*0.179*	*0.000*	*0.227*	*0.148*
pH 9	*0.187*	*0.638*	*0.000*	*0.269*	*0.000*

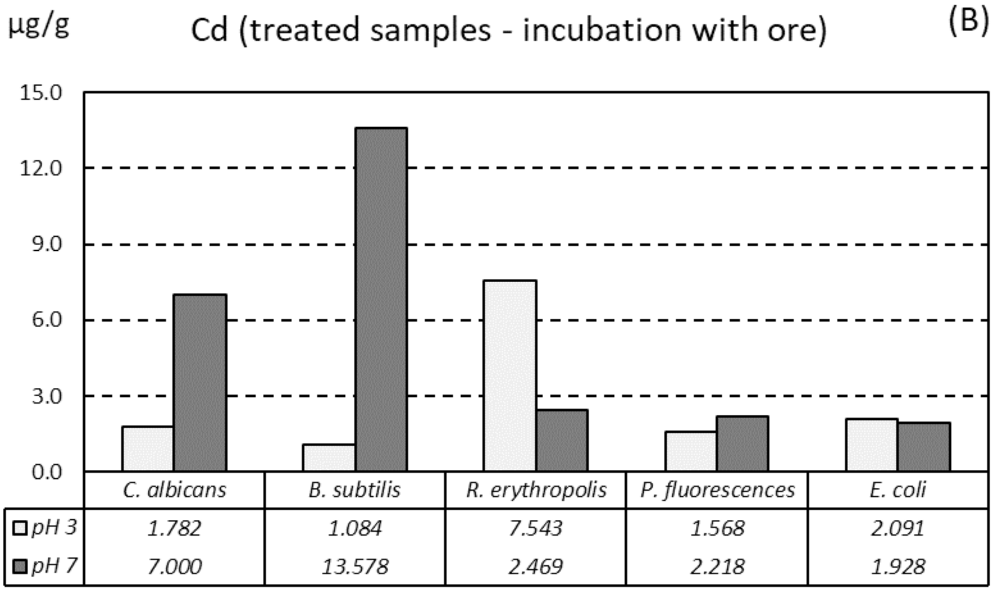

	C. albicans	*B. subtilis*	*R. erythropolis*	*P. fluorescences*	*E. coli*
pH 3	*1.782*	*1.084*	*7.543*	*1.568*	*2.091*
pH 7	*7.000*	*13.578*	*2.469*	*2.218*	*1.928*

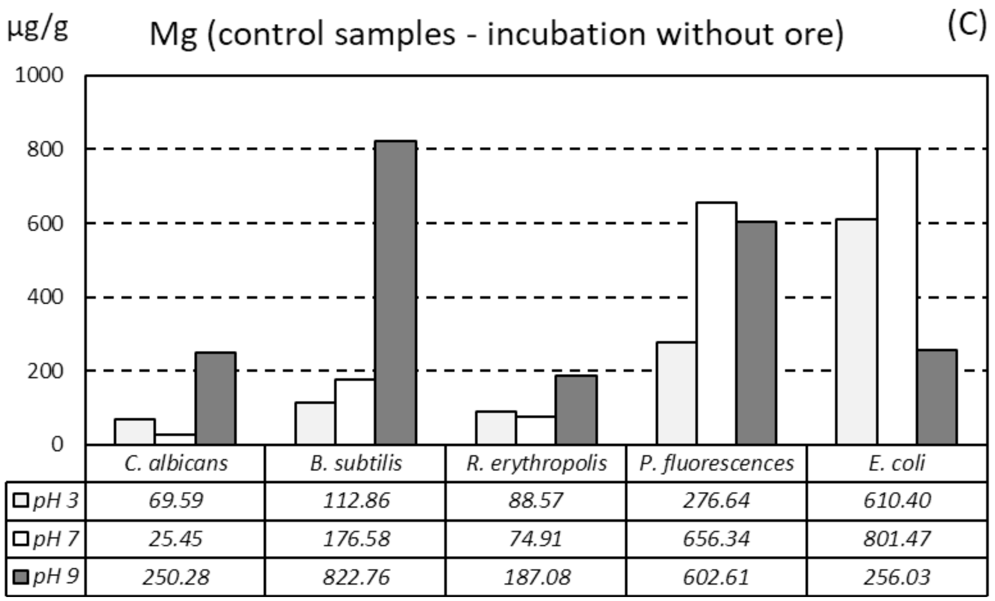

	C. albicans	B. subtilis	R. erythropolis	P. fluorescences	E. coli
pH 3	*69.59*	*112.86*	*88.57*	*276.64*	*610.40*
pH 7	*25.45*	*176.58*	*74.91*	*656.34*	*801.47*
pH 9	*250.28*	*822.76*	*187.08*	*602.61*	*256.03*

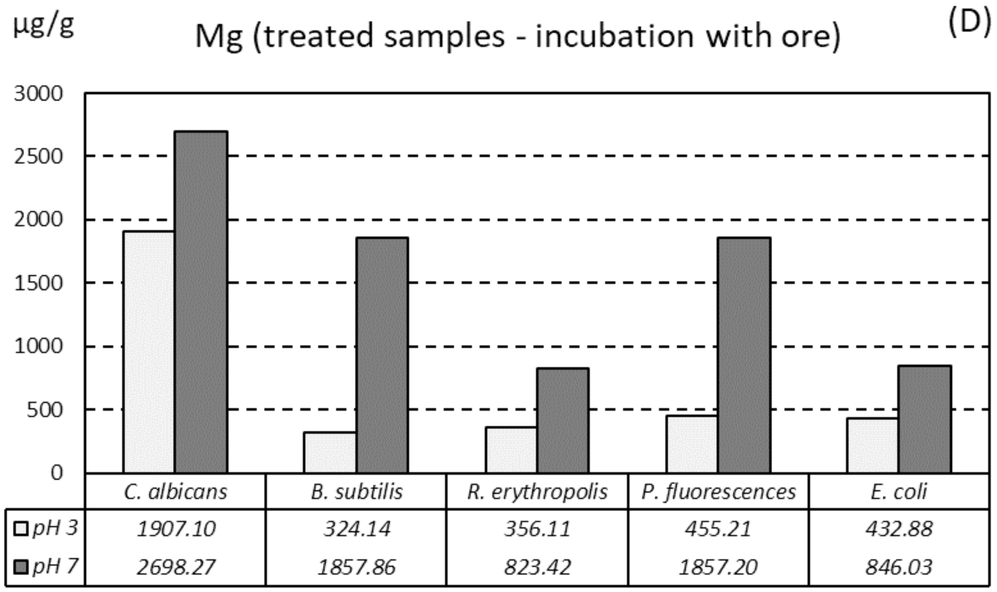

	C. albicans	B. subtilis	R. erythropolis	P. fluorescences	E. coli
pH 3	*1907.10*	*324.14*	*356.11*	*455.21*	*432.88*
pH 7	*2698.27*	*1857.86*	*823.42*	*1857.20*	*846.03*

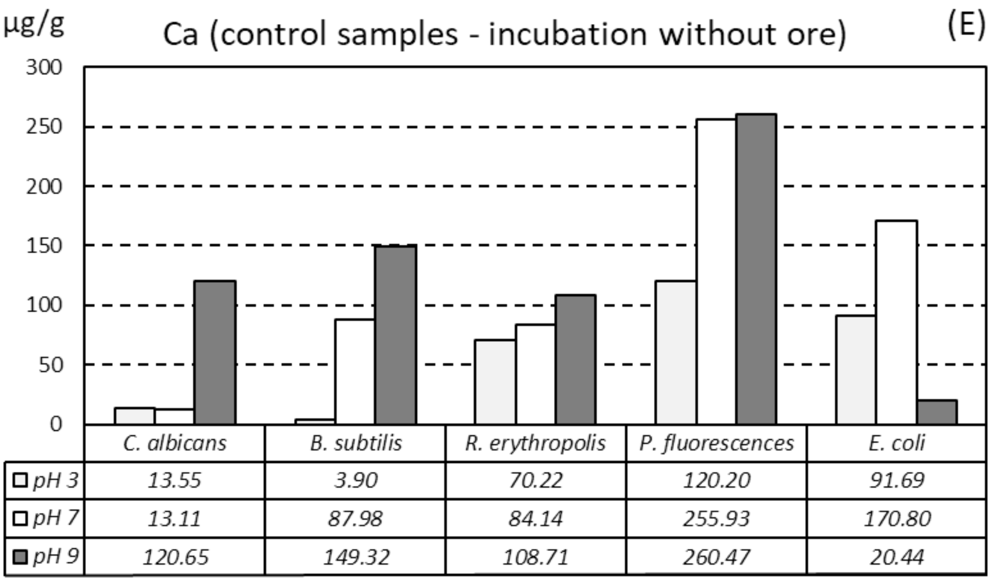

	C. albicans	*B. subtilis*	*R. erythropolis*	*P. fluorescences*	*E. coli*
pH 3	*13.55*	*3.90*	*70.22*	*120.20*	*91.69*
pH 7	*13.11*	*87.98*	*84.14*	*255.93*	*170.80*
pH 9	*120.65*	*149.32*	*108.71*	*260.47*	*20.44*

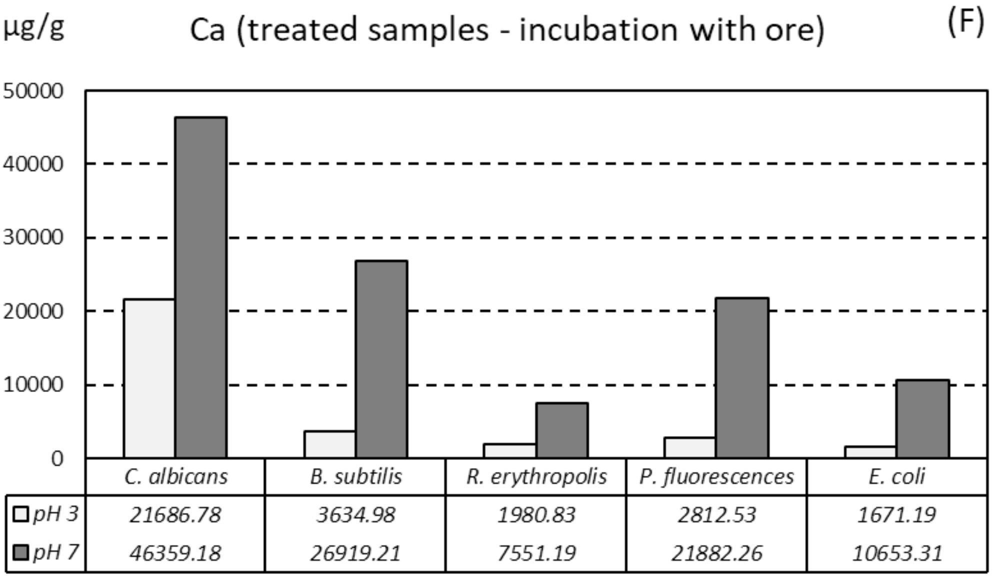

	C. albicans	*B. subtilis*	*R. erythropolis*	*P. fluorescences*	*E. coli*
pH 3	*21686.78*	*3634.98*	*1980.83*	*2812.53*	*1671.19*
pH 7	*46359.18*	*26919.21*	*7551.19*	*21882.26*	*10653.31*

Fig. S2. SEM images of *B. subtilis* on apatite or dolomite from Djebel Onk ore, after incubation at pH 4 (at 28 °C for 20 min). Red circles - areas which were magnified and showed in the second column.

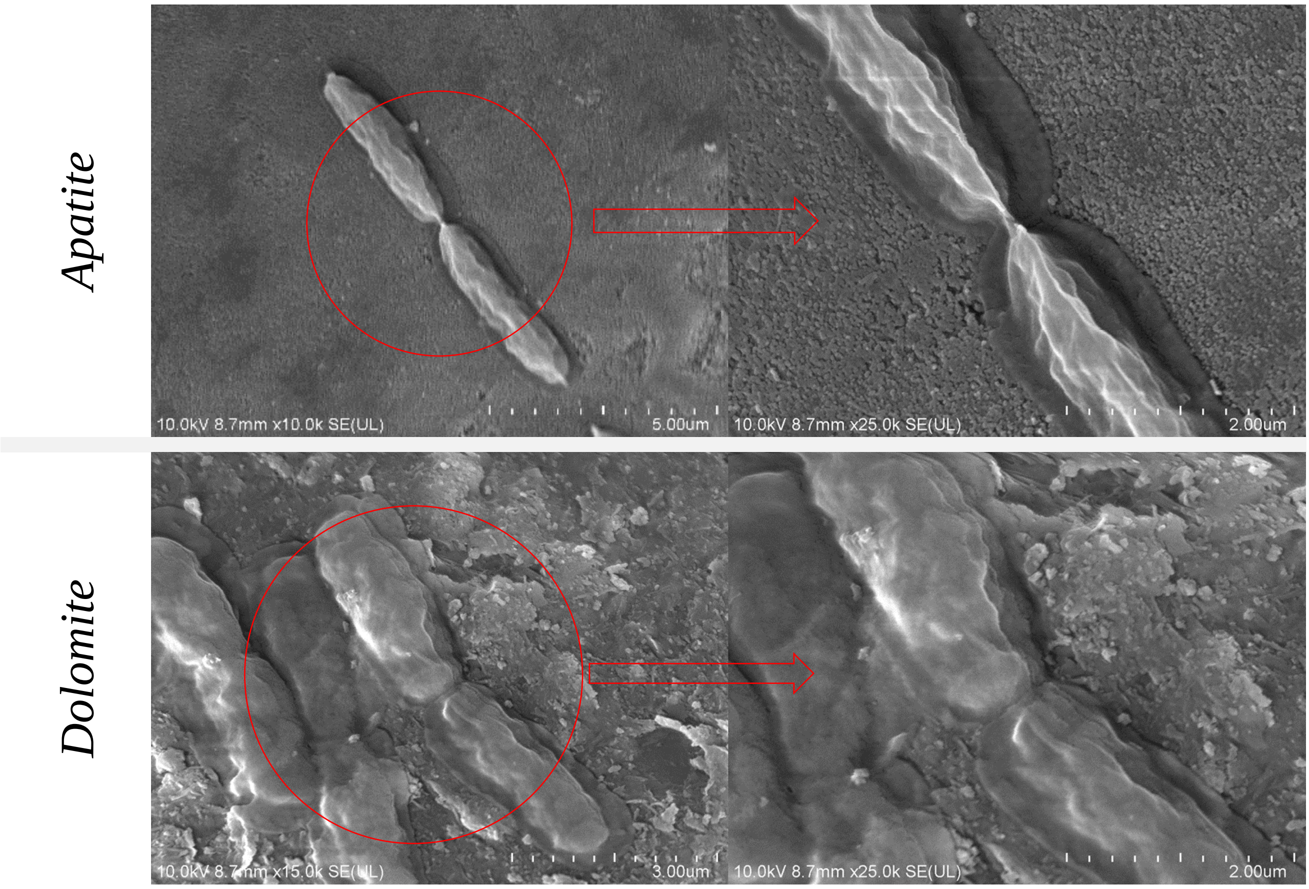

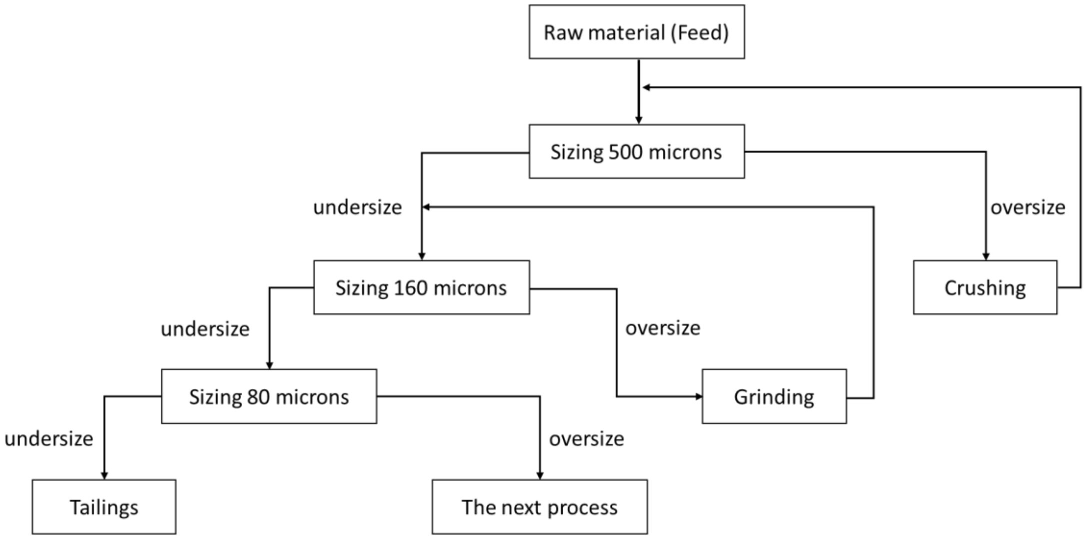

Fig S3. Technological procedures conducting for preparation 80-160 μm fraction.

Table S1. The amount of Cd, Mg, and Ca (mean ± SD) leached out from the sample of ore during 20 min of incubation at various pH.

pH	Cd [µg·L^{-1}]		Mg [mg·L^{-1}]		Ca [mg·L^{-1}]	
	mean	SD	mean	SD	mean	SD
4	3.224	0.865	1.355	0.090	10.852	0.334
6	3.407	0.857	1.224	0.064	9.590	0.275
8	1.860	0.219	1.125	0.001	8.856	0.011
10	1.474	0.037	0.997	0.043	8.009	0.360
12	1.376	0.466	0.678	0.021	4.959	0.428

Fig. S1_bis. Cd, Mg, and Ca accumulation (mean ± SD; μg·g[-1]) in biomass (*B. subtilis* - A, C, E, and *C. albicans* - B, D, F) after incubation with phosphate ore up to 70 min. The same letter indicate no significant differences among time-groups (ANOVA, LSD; $p<0.05$).

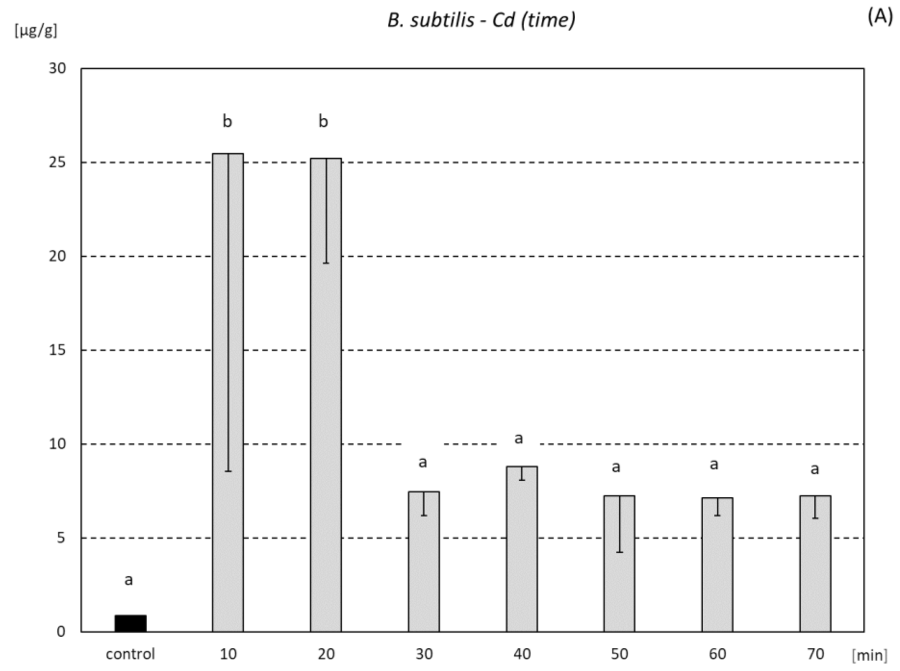

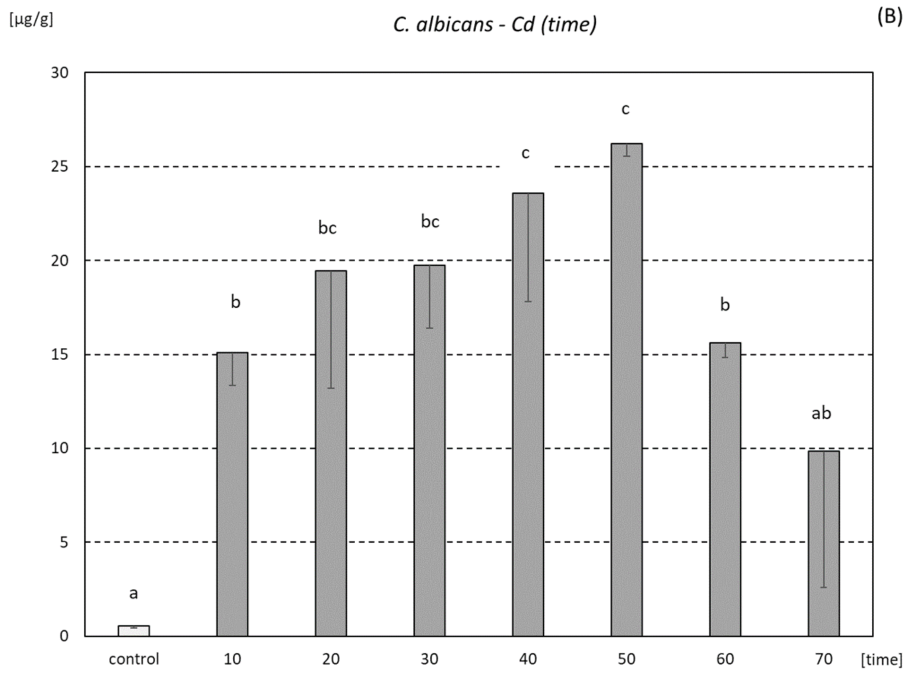

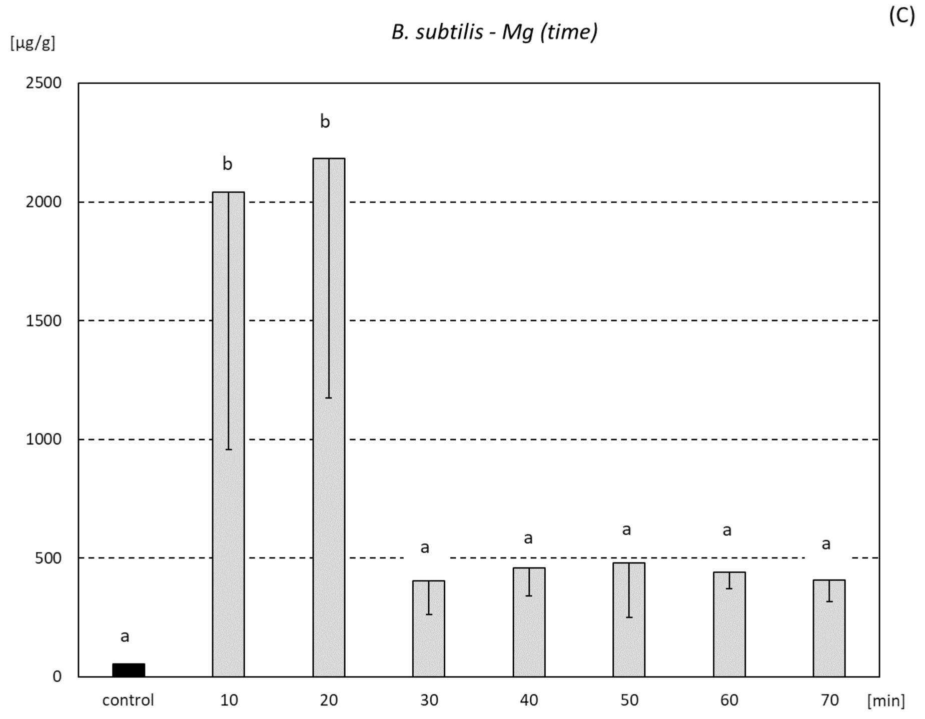

(C)
B. subtilis - Mg (time)
[µg/g]
2500
2000
1500
1000
500
0
b
b
a
a
a
a
a
a
control
10
20
30
40
50
60
70
[min]

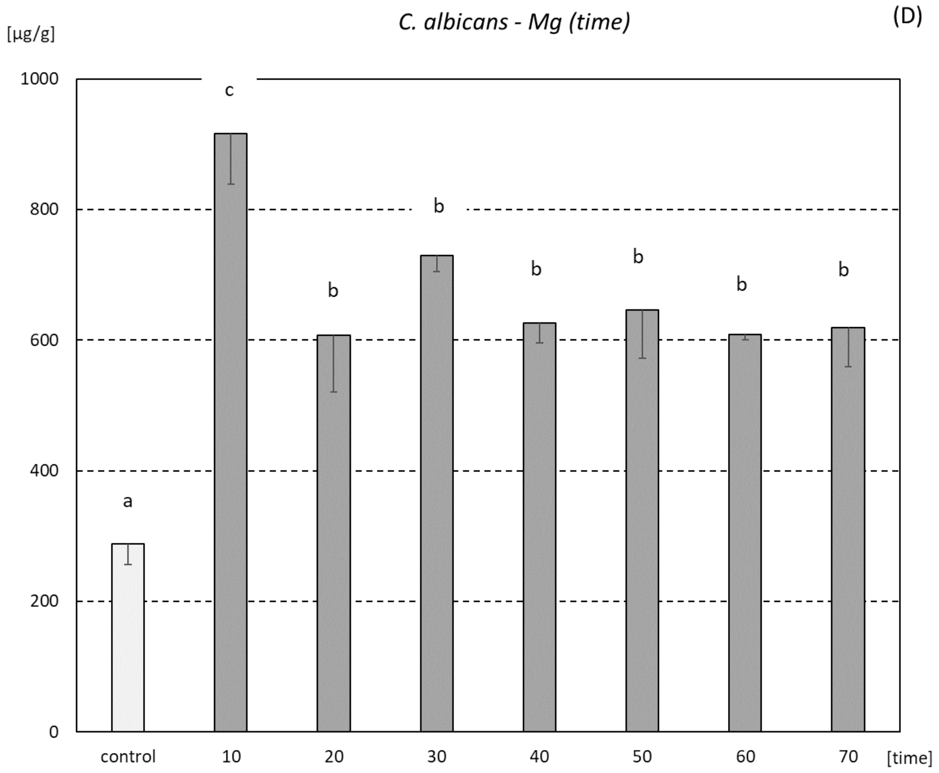

(D)
C. albicans - Mg (time)
[µg/g]
1000
800
600
400
200
0
c
b
b
b
b
b
b
a
control
10
20
30
40
50
60
70
[time]

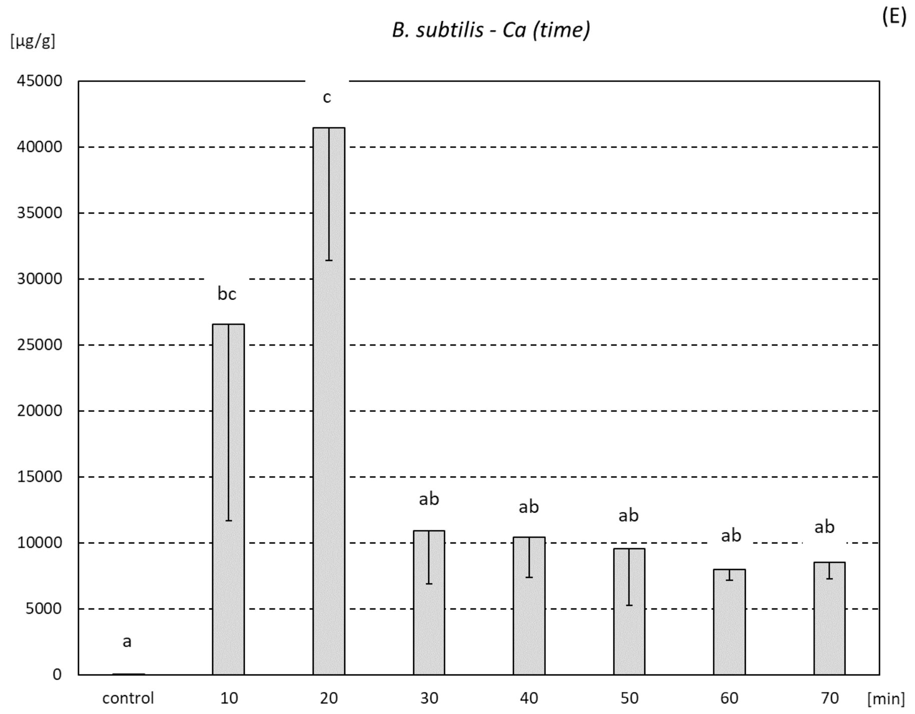
(E)
B. subtilis - Ca (time)
[µg/g]
45000
40000
35000
30000
25000
20000
15000
10000
5000
0
a
bc
c
ab
ab
ab
ab
ab
control
10
20
30
40
50
60
70
[min]

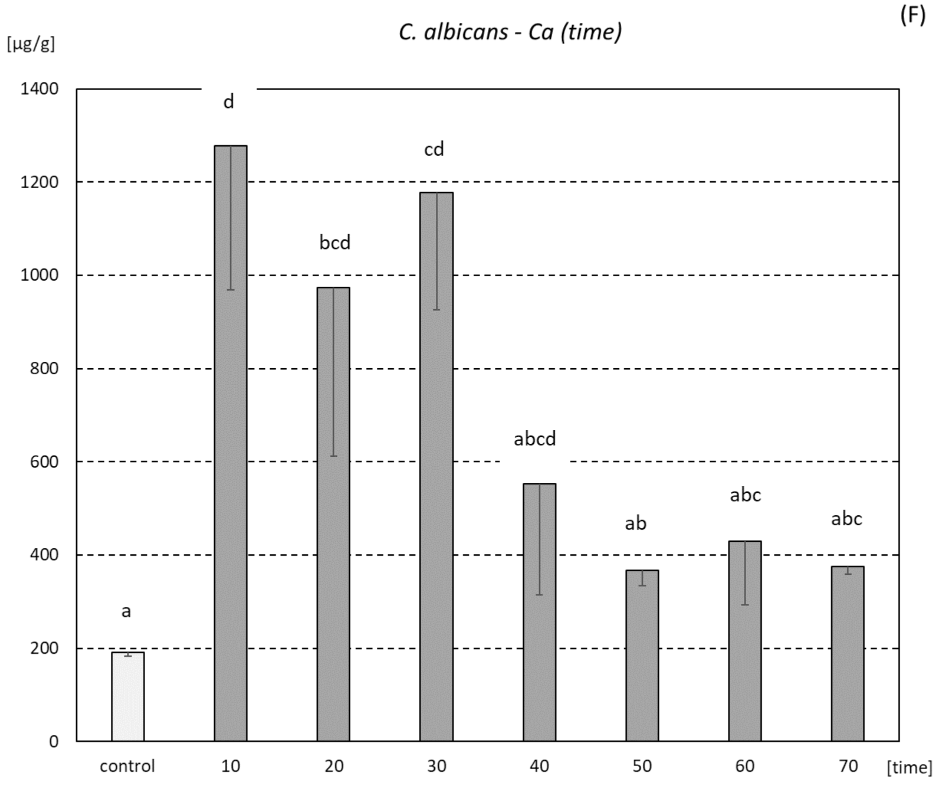
(F)
C. albicans - Ca (time)
[µg/g]
1400
1200
1000
800
600
400
200
0
a
d
bcd
cd
abcd
ab
abc
abc
control
10
20
30
40
50
60
70
[time]

Fig. S2_bis. Cd, Mg, and Ca accumulation (mean ± SD; μg·g^{-1}) in biomass (*B. subtilis* - A, C, E, and *C. albicans* - B, D, F) after incubation with phosphate ore at different pH. The same letter indicate no significant differences among pH-groups (ANOVA, LSD; p<0.05).

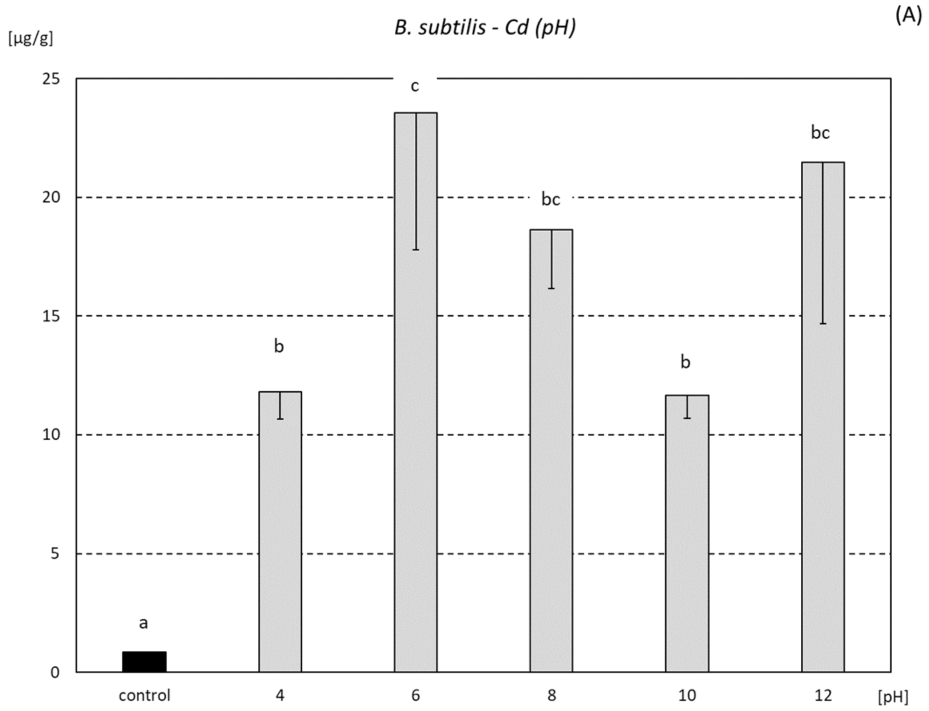

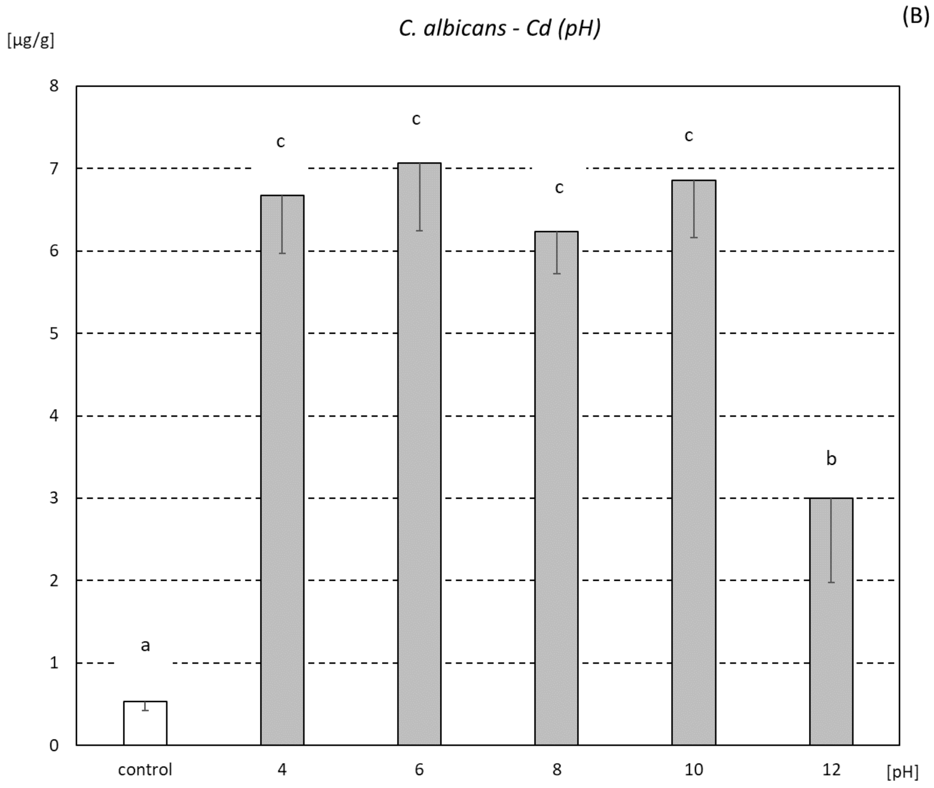

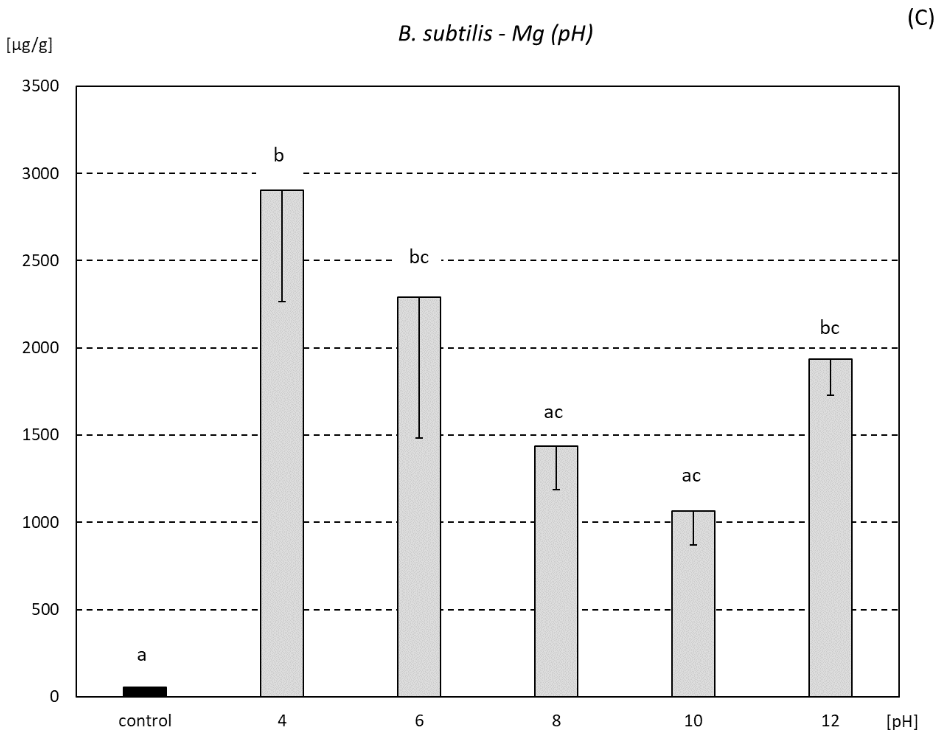
(C)
B. subtilis - Mg (pH)
[μg/g]
3500
3000
2500
2000
1500
1000
500
0
b
bc
ac
ac
bc
a
control
4
6
8
10
12
[pH]

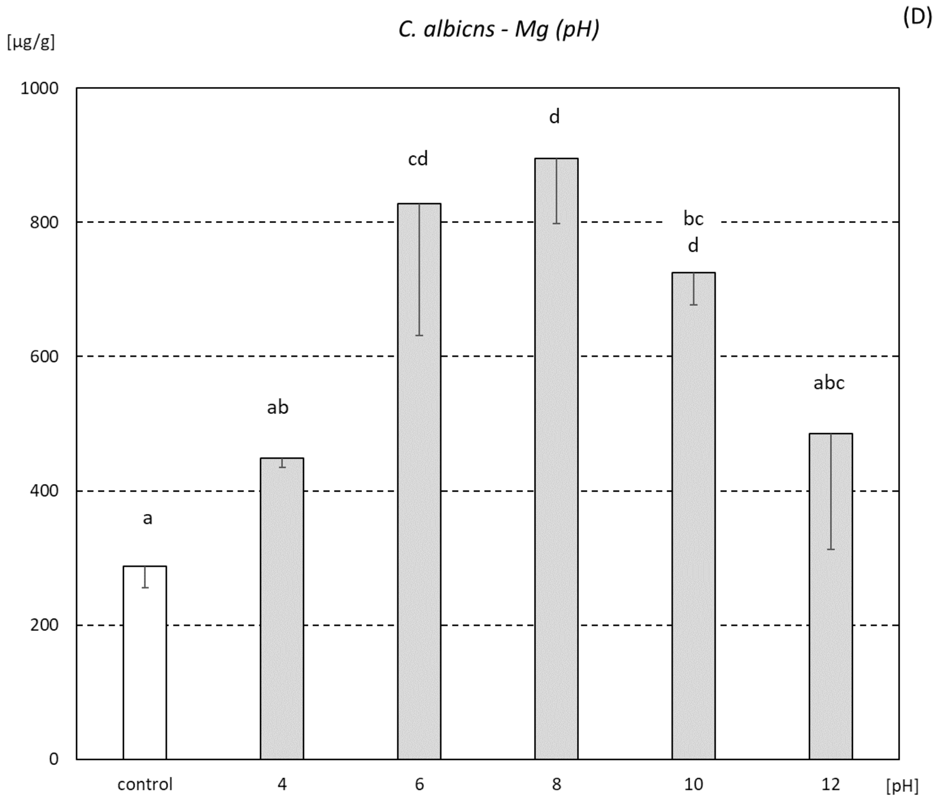
(D)
C. albicns - Mg (pH)
[μg/g]
1000
800
600
400
200
0
d
cd
bc
d
abc
ab
a
control
4
6
8
10
12
[pH]

(E)

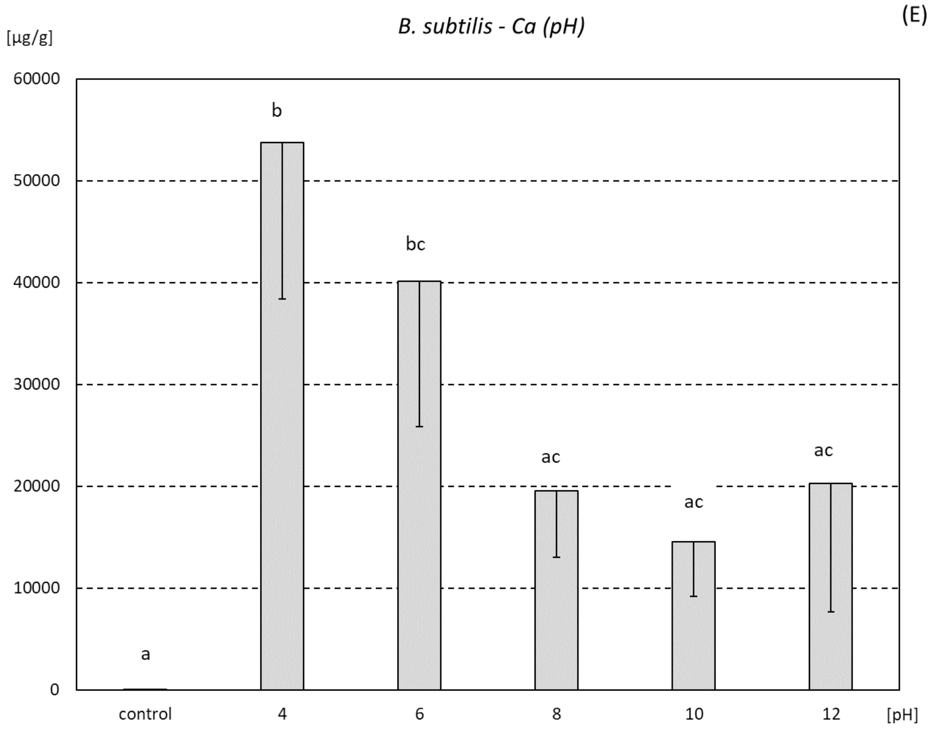

B. subtilis - Ca (pH)
[µg/g]
60000
50000
40000
30000
20000
10000
0
b
bc
ac
ac
ac
a
control
4
6
8
10
12
[pH]

(F)

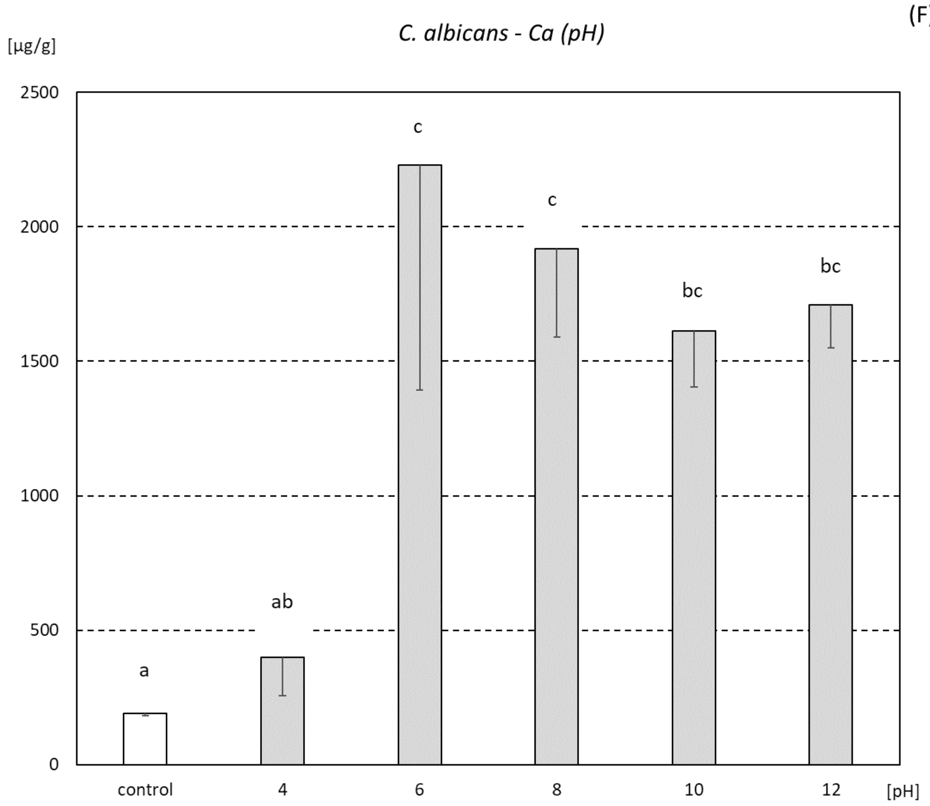

C. albicans - Ca (pH)
[µg/g]
2500
2000
1500
1000
500
0
c
c
bc
bc
ab
a
control
4
6
8
10
12
[pH]

Fig. S6. SEM images of apatite or dolomite from Djebel Onk ore, showing various microorganism adsorption onto the minerals surface. Abbreviations: microorganism strains: *Candida albicans* (**C.a**.), *Bacillus subtilis* (**B.s**.), *Rhodococcus erythropolis* CD 130 (**R.e.**), *Pseudomonas fluorescens* (**P.f.**), and *Escherichia coli* (**E.c.**) were incubated with ores at different pH (3 or 7), and at 28 °C for 20 min. Red circles - example of microorganisms.

C.a. on apatite (pH 3)

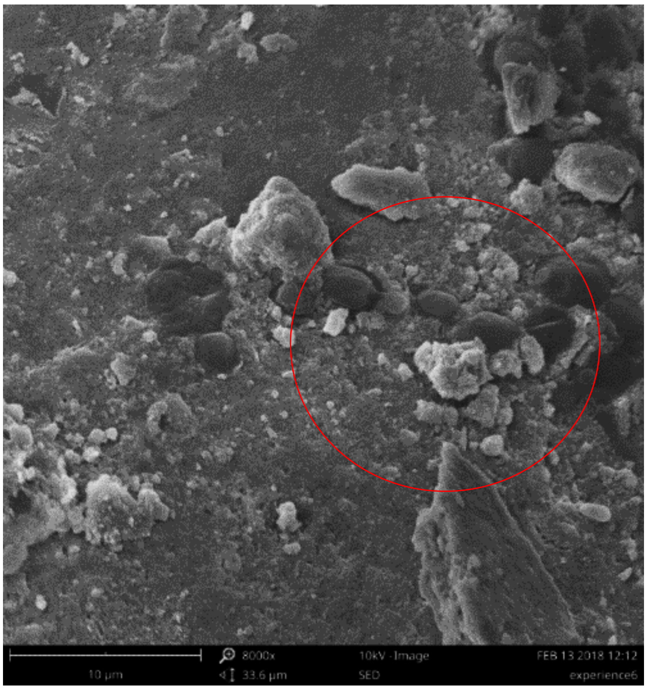

C.a. on apatite (pH 7)

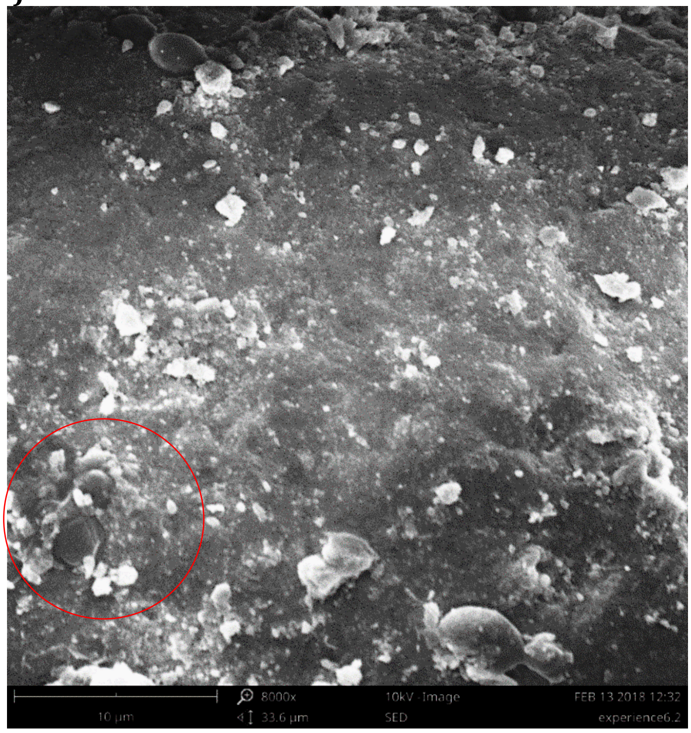

C.a. on dolomite (pH 3)

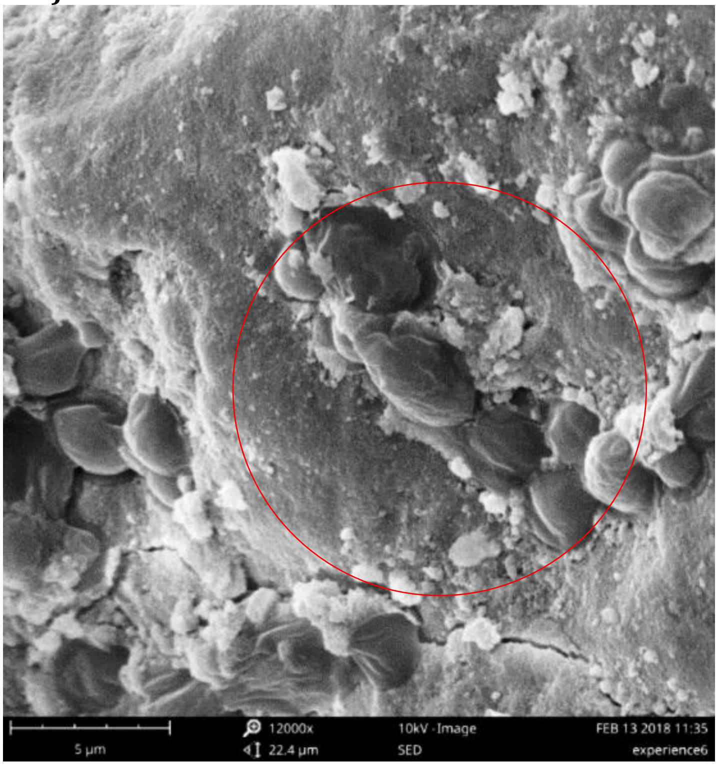

C.a. on dolomite (pH 7)

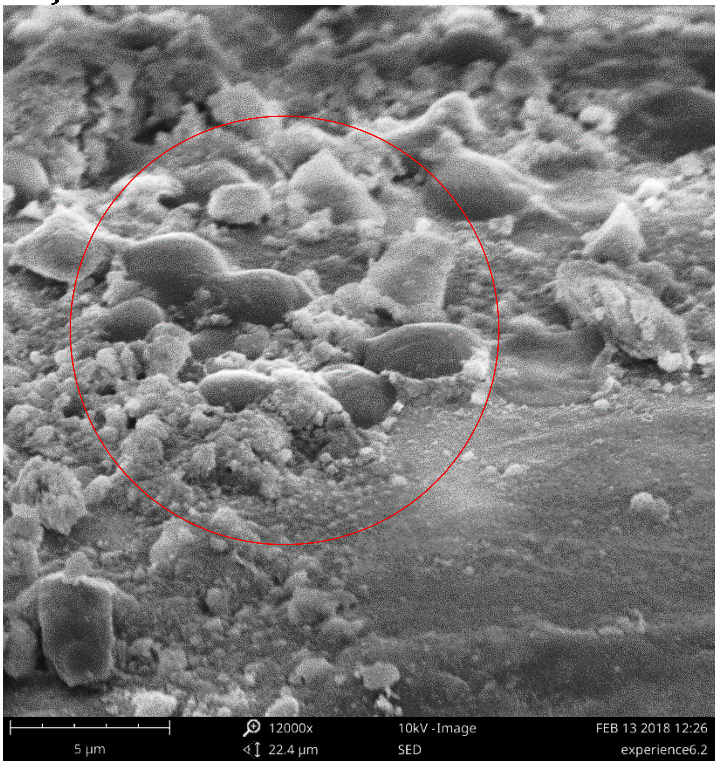

B.s. on apatite (pH 3)

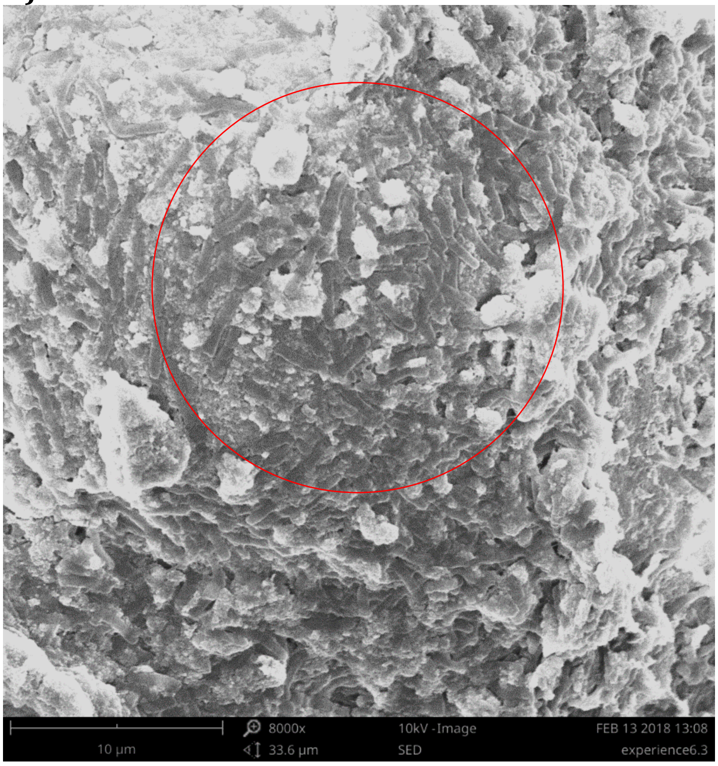

B.s. on apatite (pH 7) - bacteria not visible (very weak adhesion)

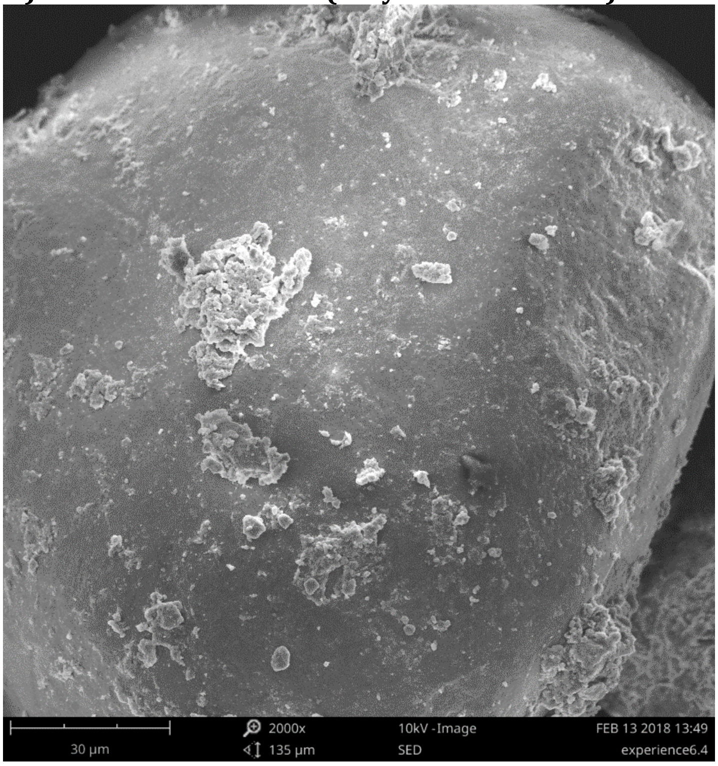

B.s. on dolomite (pH 3)

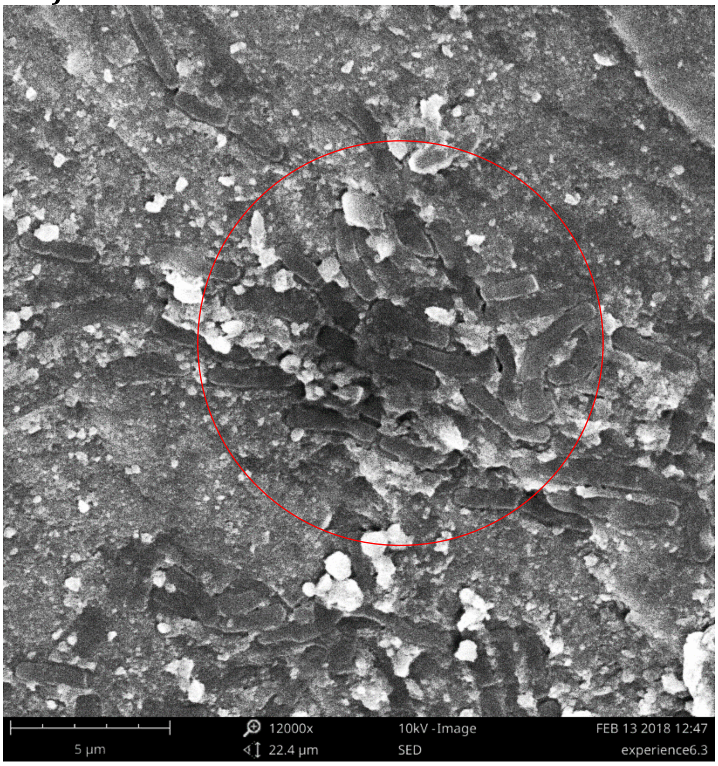

B.s. on dolomite (pH 7) - bacteria not visible (very weak adhesion)

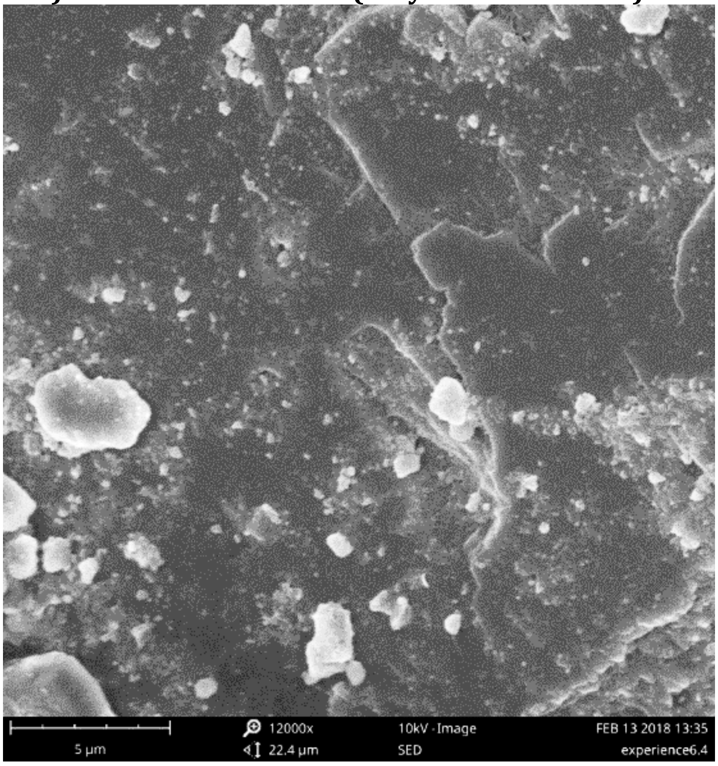

R.e. on apatite (pH 3)

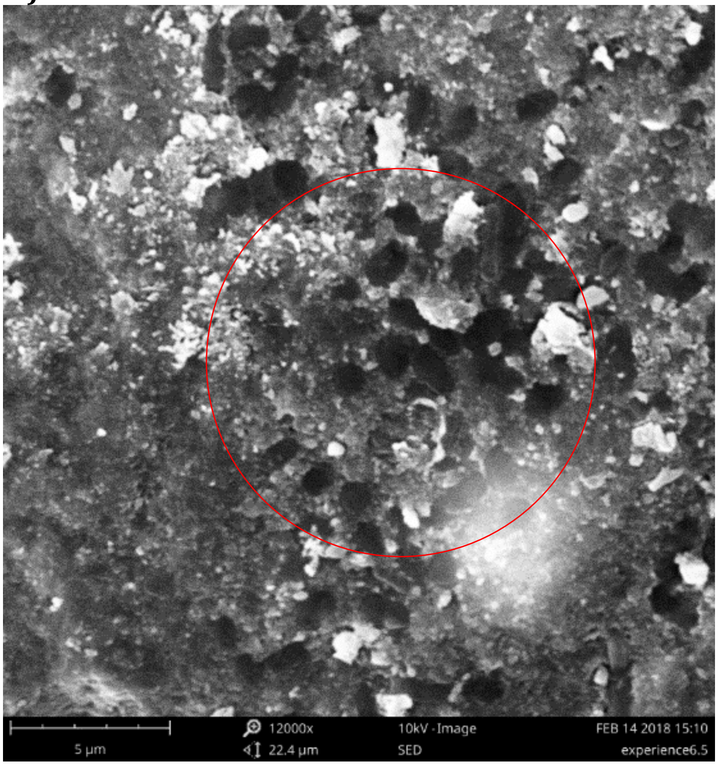

R.e. on apatite (pH 7)

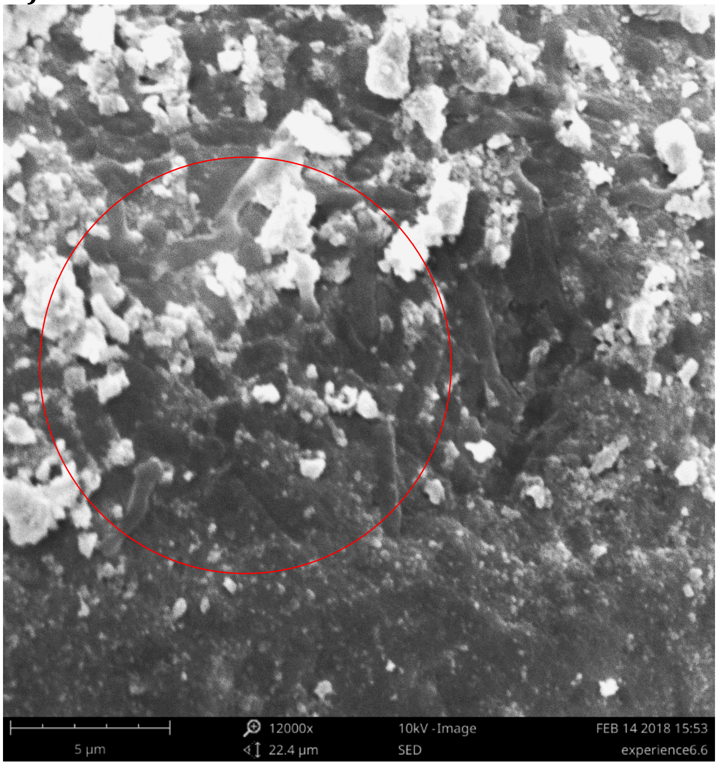

R.e. on dolomite (pH 3) - bacteria not visible (very weak adhesion)

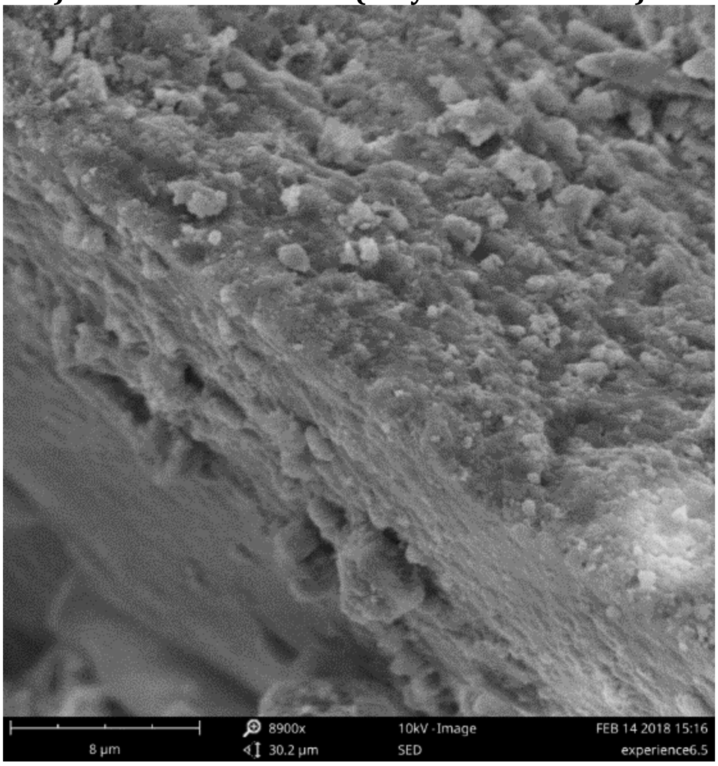

R.e. on dolomite (pH 7)

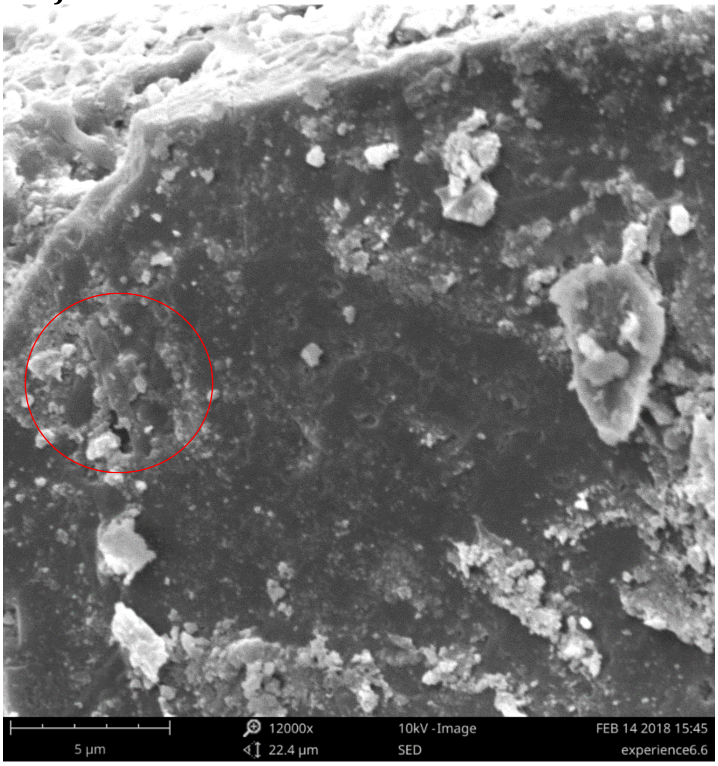

P.f. on apatite (pH 3)

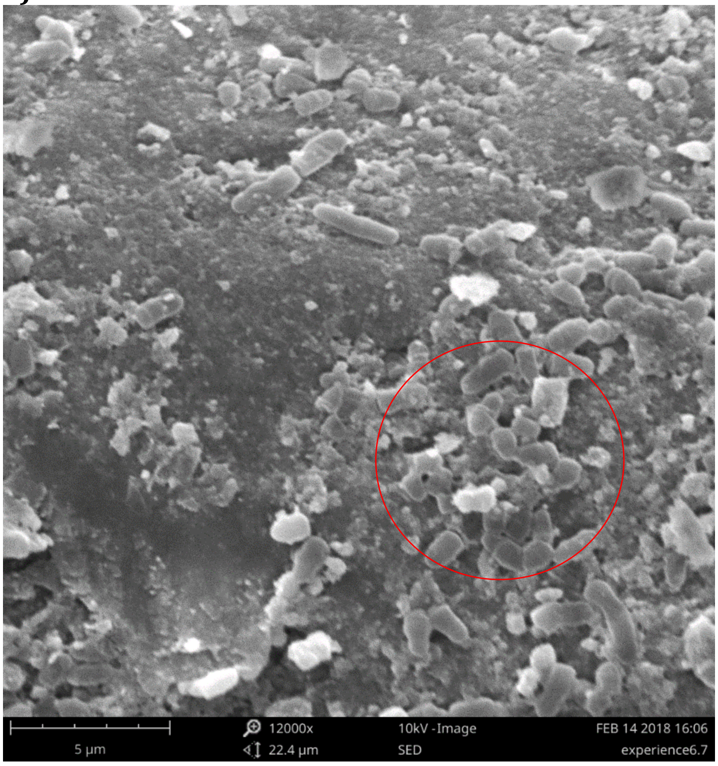

P.f. on apatite (pH 7)

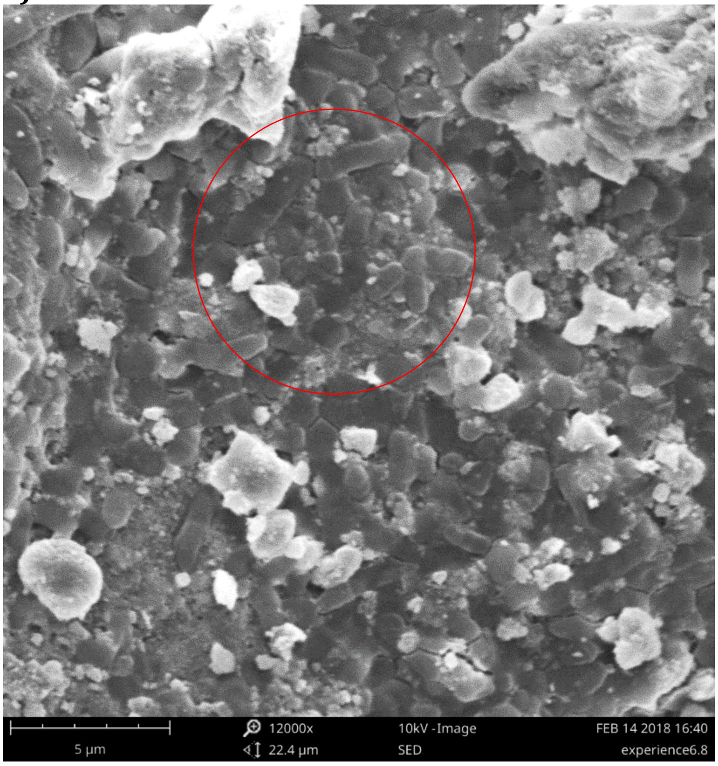

P.f. on dolomite (pH 3) - bacteria not visible (very weak adhesion)

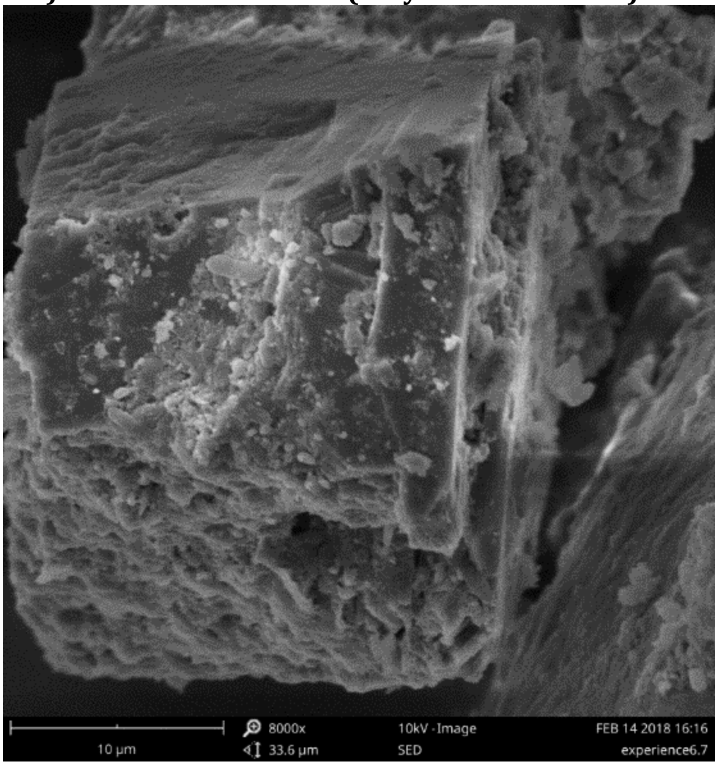

P.f. on dolomite (pH7) - bacteria not visible (very weak adhesion)

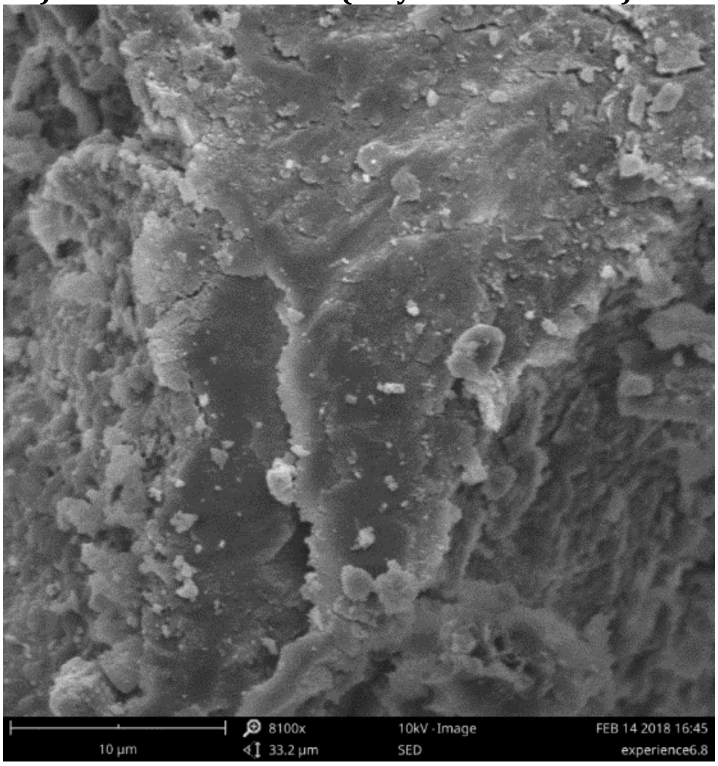

E.c. on apatite (pH3)

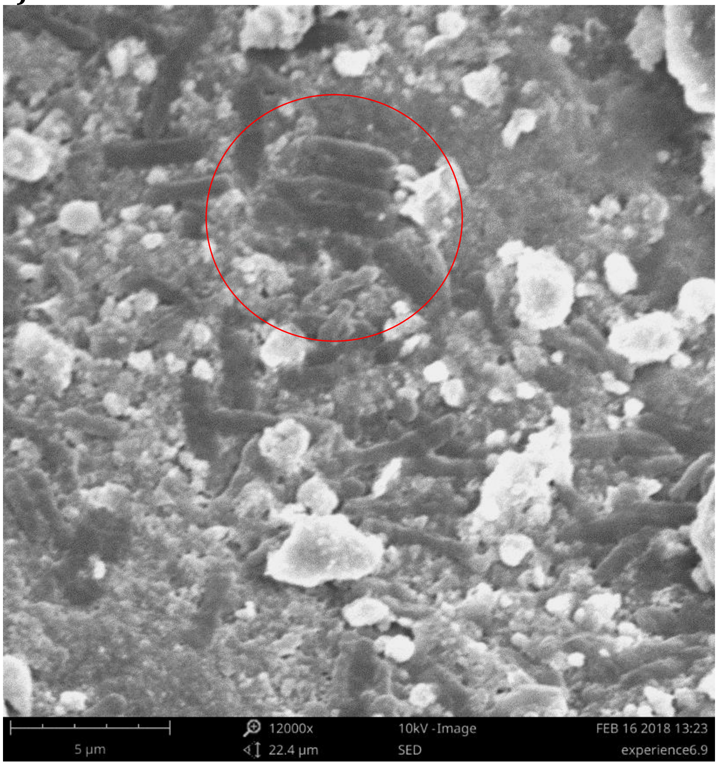

E.c. on apatite (pH7)

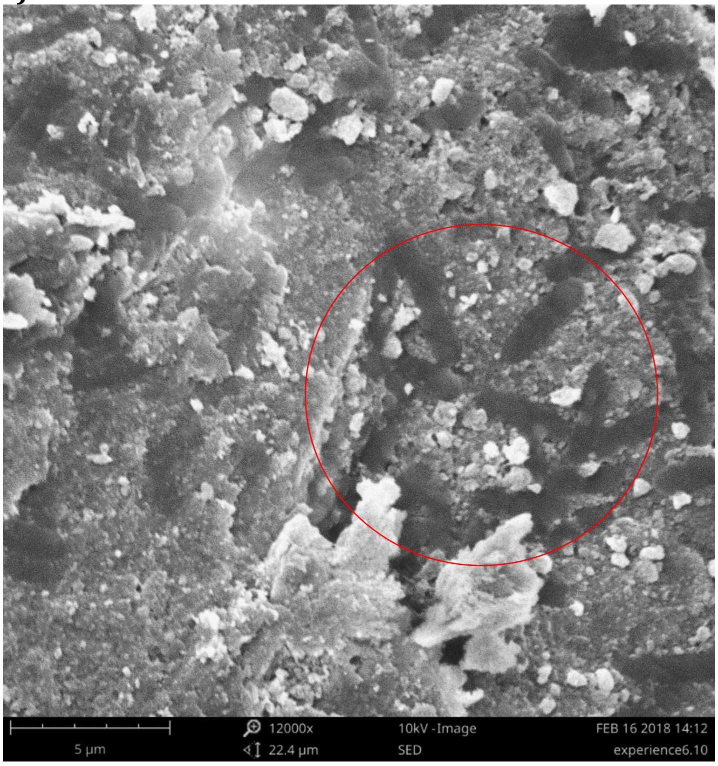

E.c on dolomite (pH3)

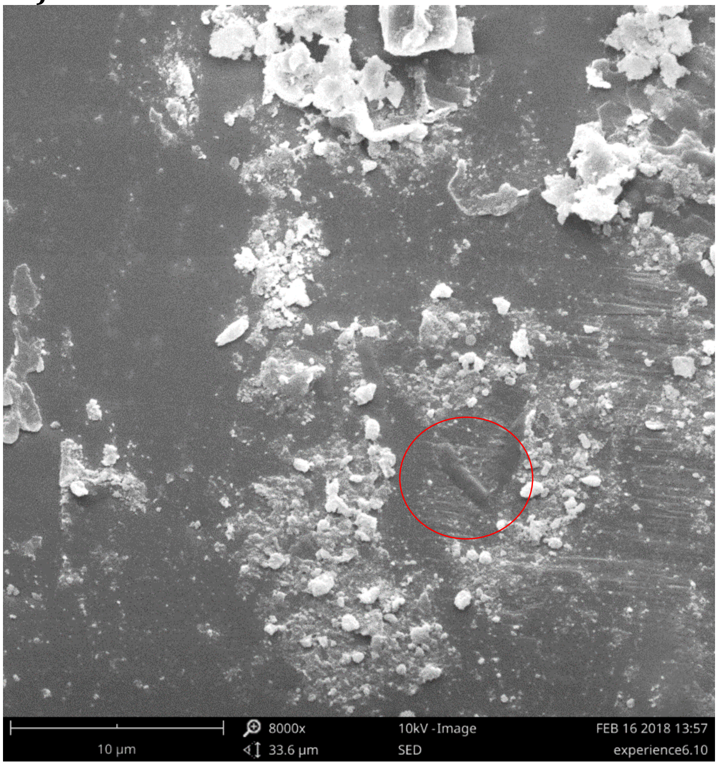

E.c. on dolomite (pH7) - bacteria not visible (very weak adhesion)

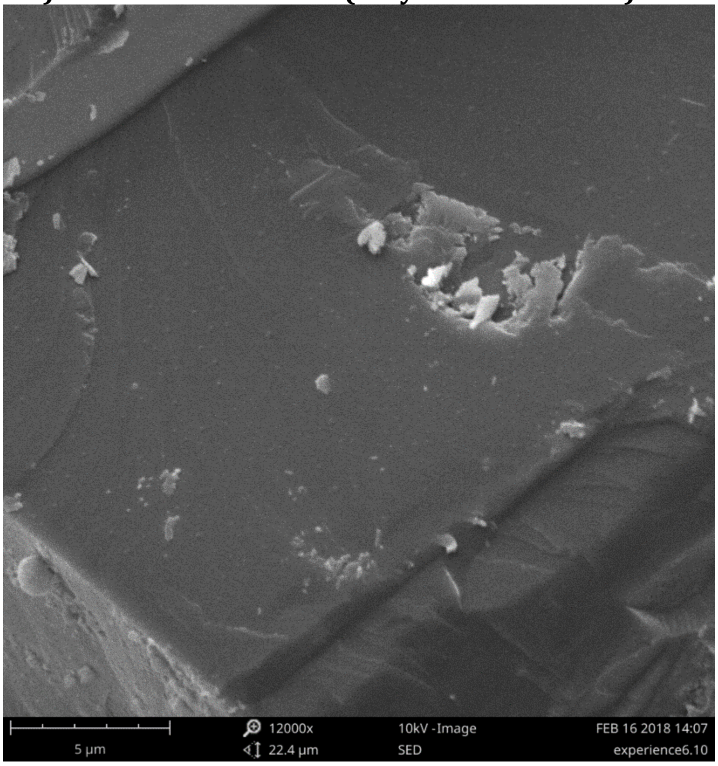

Fig. S7. SEM images of apatite and dolomite from Djebel Onk ore, showing *B. subtilis* adsorption onto the minerals surface after incubation at pH 4 or 10 (at 28 °C for 20 min). Red circles - areas which were magnified and showed in the second column, *Bacillus subtilis* (**B.s.**)

B.s. on apatite (pH 4)

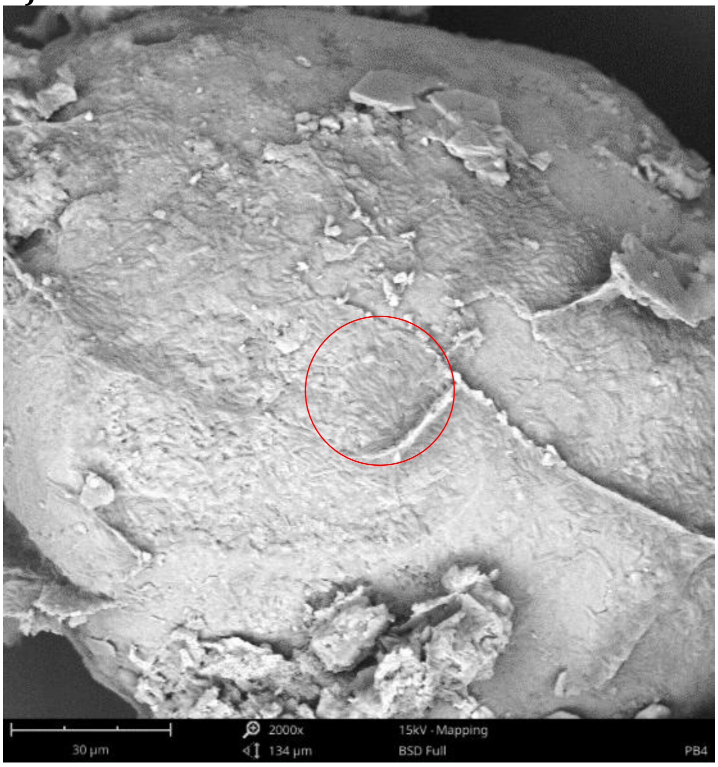

B.s. on apatite (pH 4) - enlarged

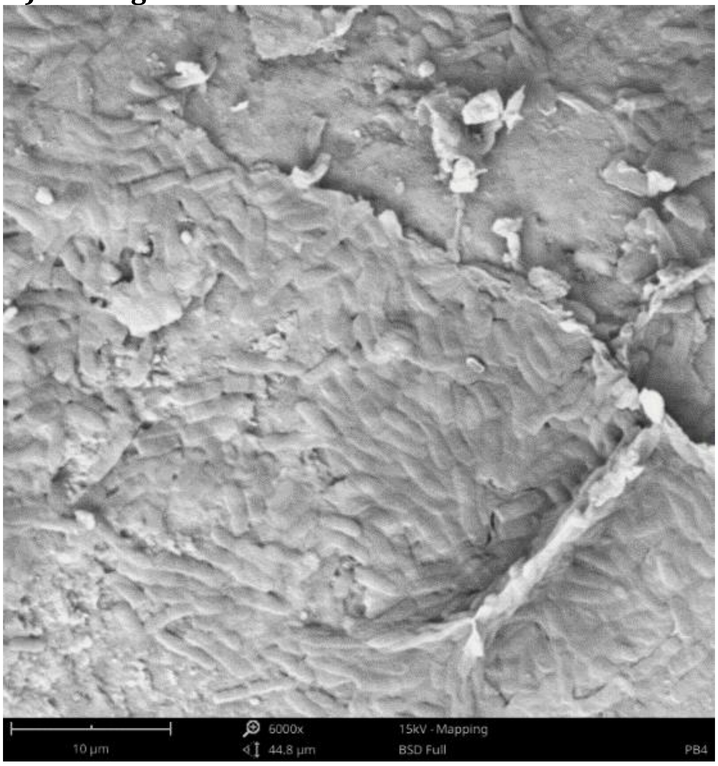

B.s. on apatite (pH 4)

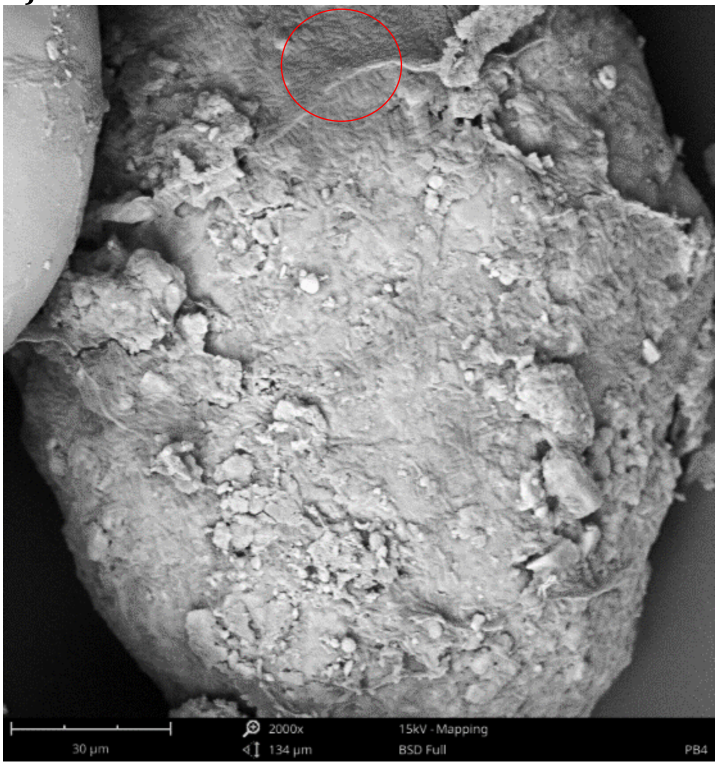

B.s. on apatite (pH 4) - enlarged

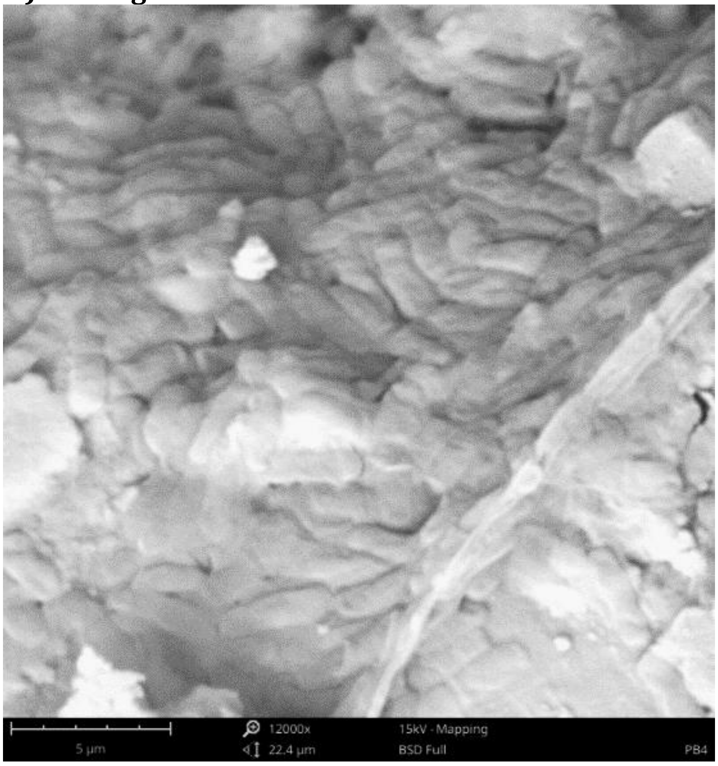

B.s. on apatite (pH 10)

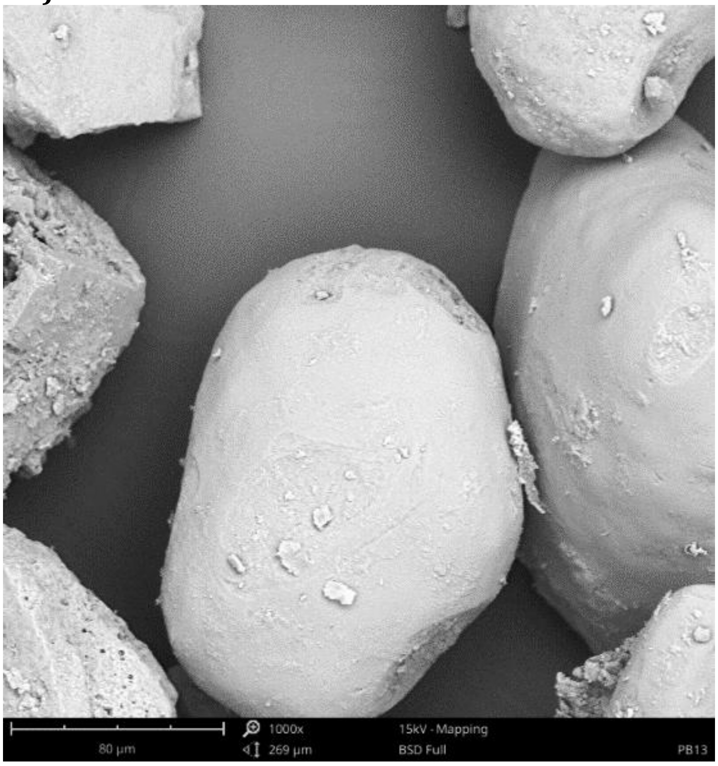

B.s. on apatite (pH 10)

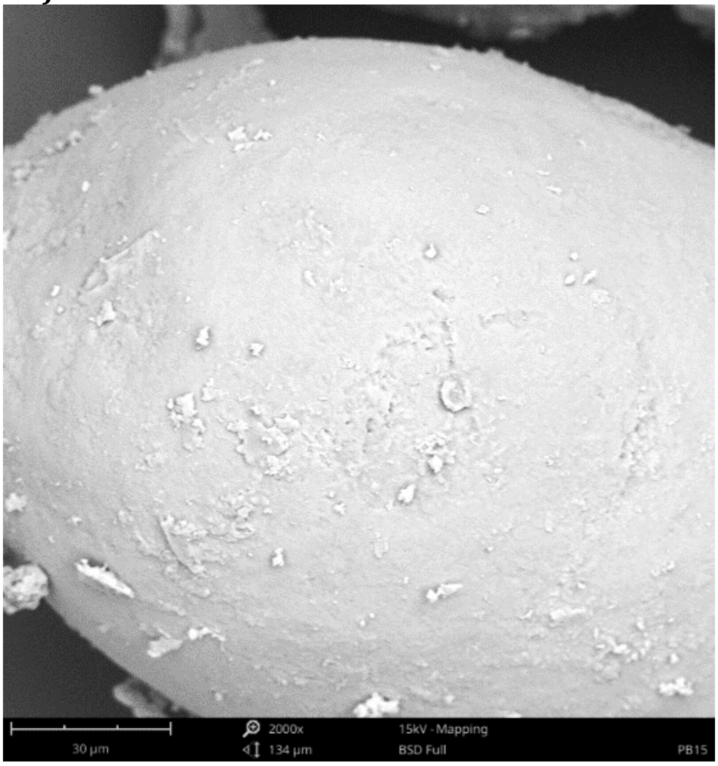

B.s. on dolomite (pH 4)

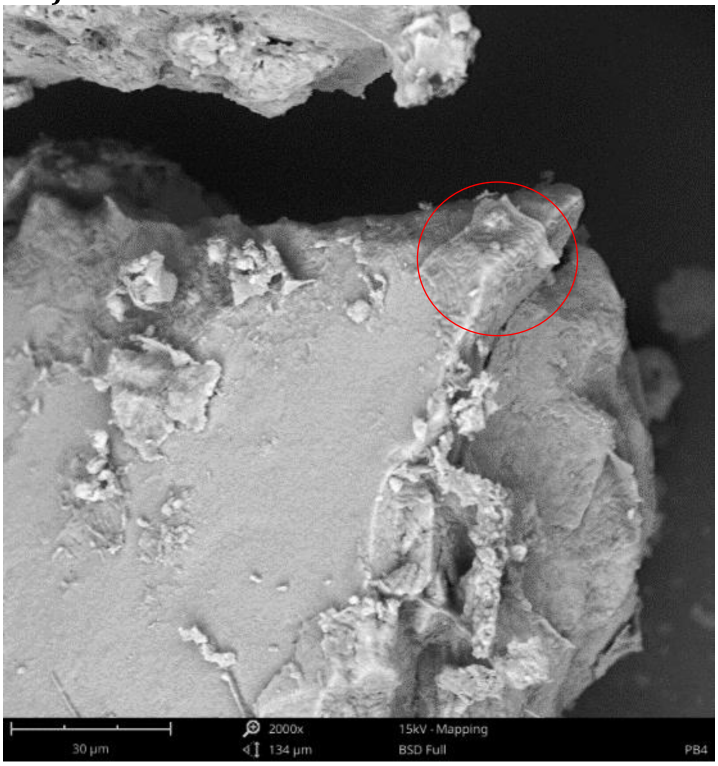

B.s. on dolomite (pH 4) - enlarged

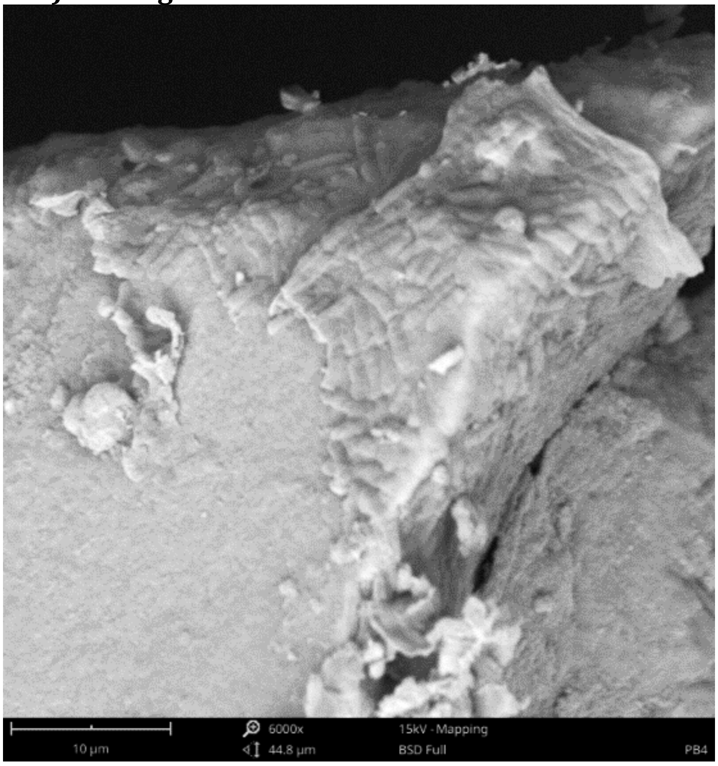

B.s. on dolomite (pH 4)

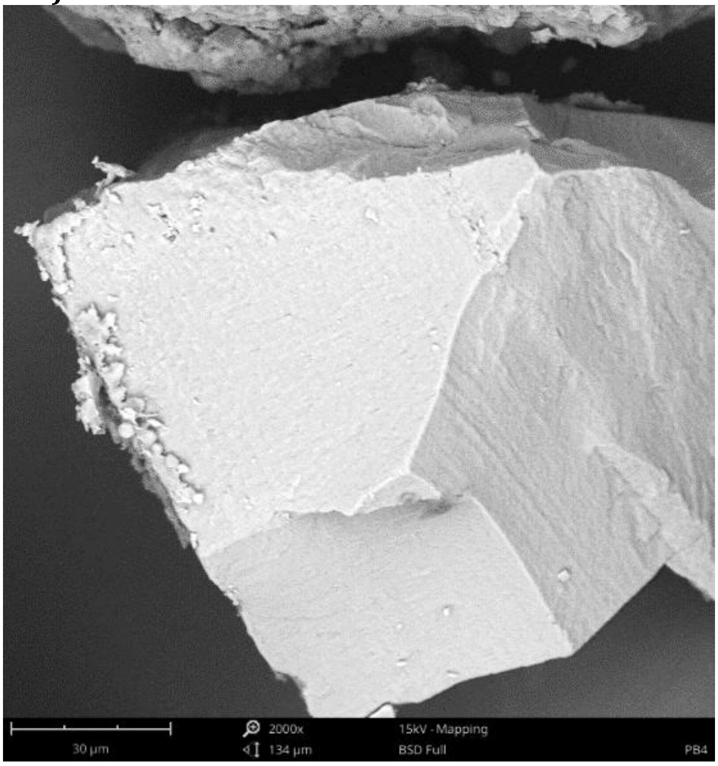

B.s. on dolomite (pH 4) - enlarged

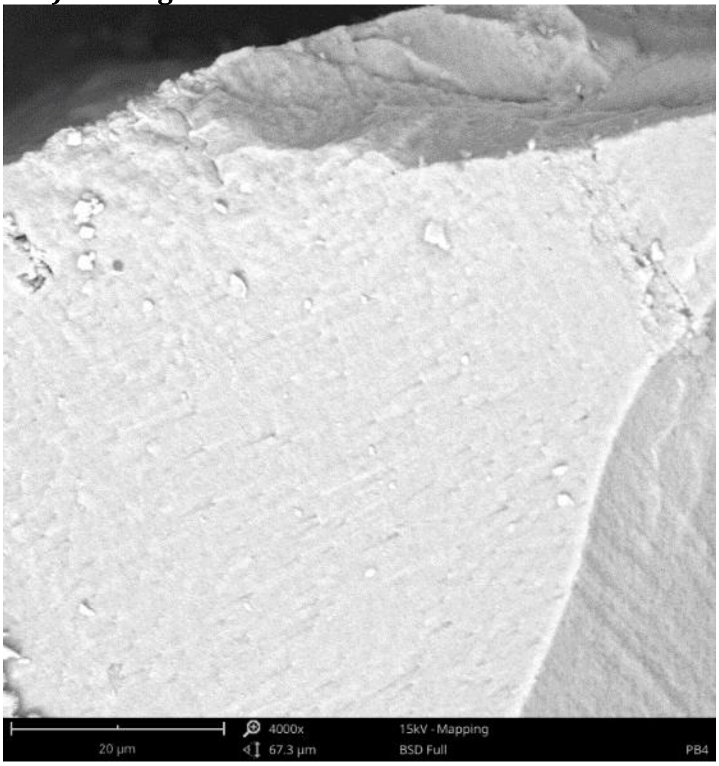

B.s. on dolomite (pH 10)

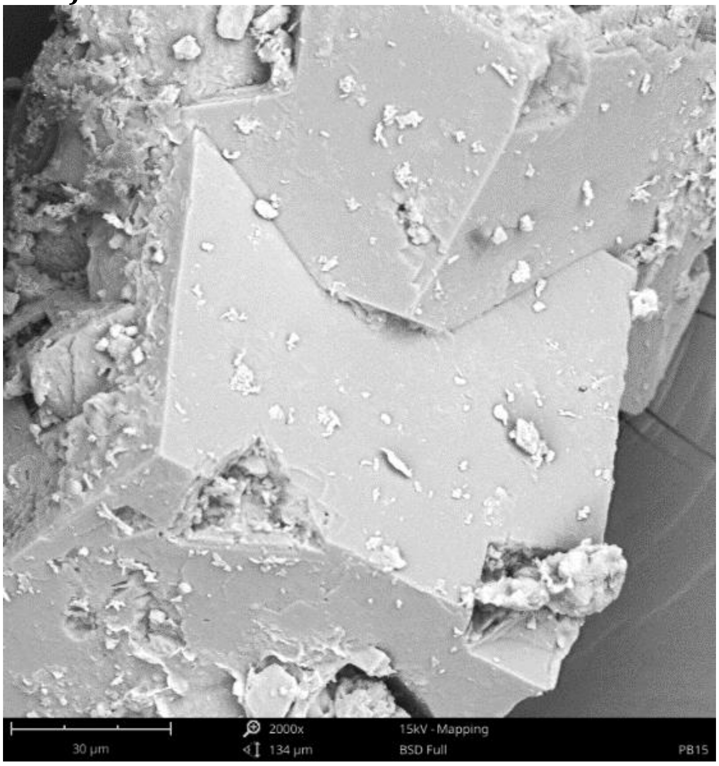

B.s. on dolomite (pH 10)

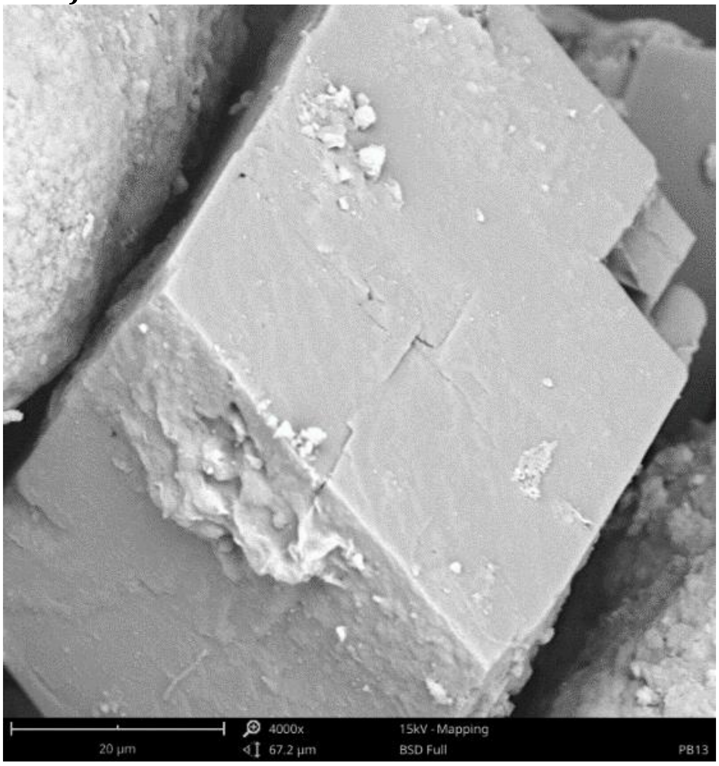

Fig. S8. SEM images of apatite and dolomite from Djebel Onk ore, showing *C. albicans* (**C.a.**) adsorption onto the minerals surface after incubation at pH 3, 5 or 9.5 (at 28 °C for 20 min).

C.a. on apatite (pH 3)

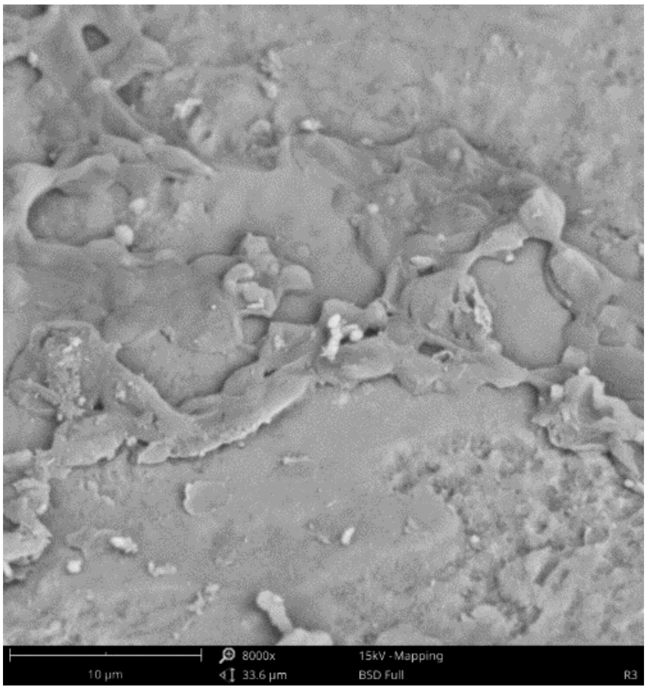

C.a. on dolomite (pH 3)

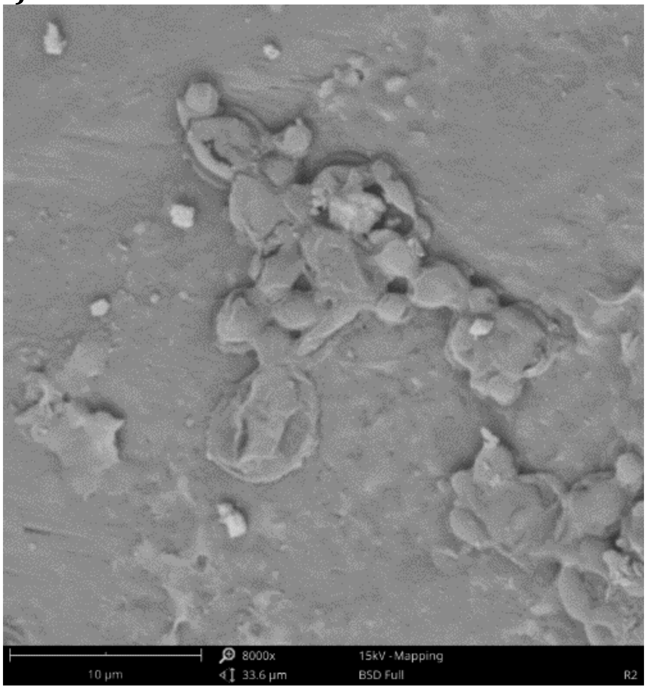

C.a. on apatite (pH 5)

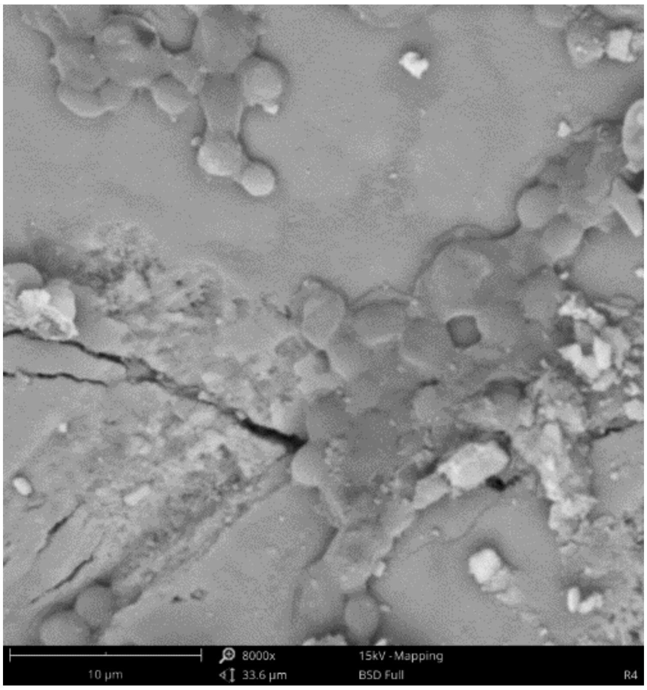

C.a. on dolomite (pH 5)

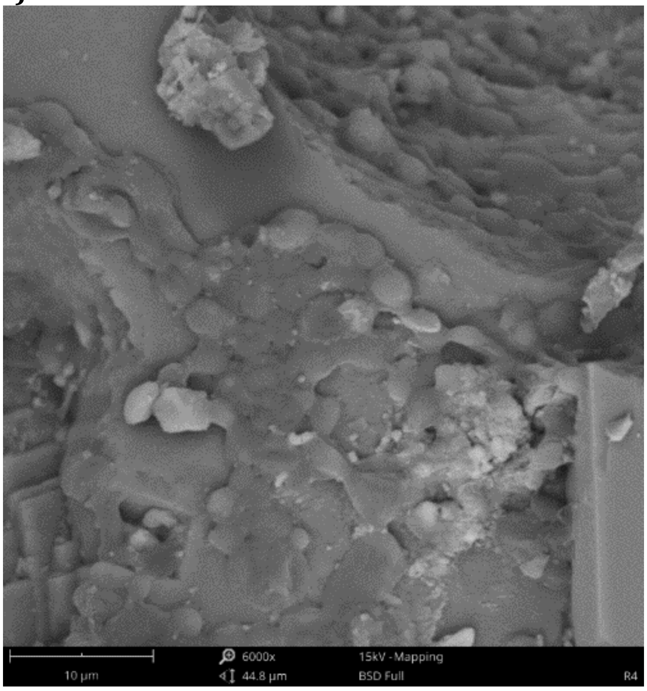

C.a. on apatite (pH 9)

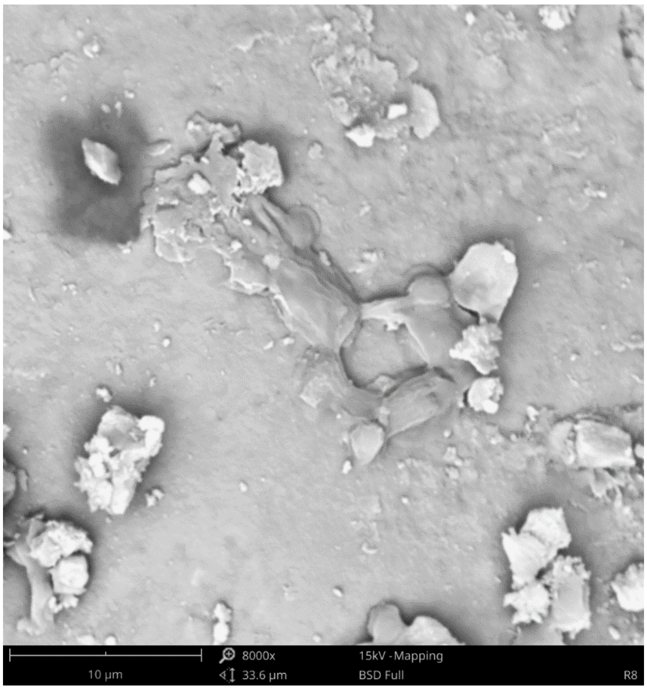

C.a. on dolomite (pH 9)

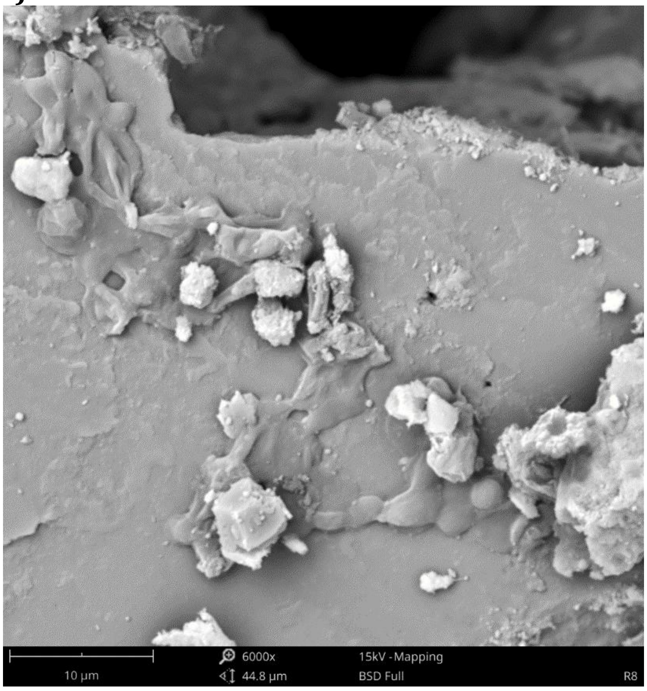

Supplement: Supplementary file 1 — Supplementary information [file 41598_2019_49406_MOESM1_ESM.pdf]
